# Development and application of versatile high density microarrays for genome-wide analysis of *Streptomyces coelicolor*: characterization of the HspR regulon

**DOI:** 10.1186/gb-2009-10-1-r5

**Published:** 2009-01-16

**Authors:** Giselda Bucca, Emma Laing, Vassilis Mersinias, Nicholas Allenby, Douglas Hurd, Jolyon Holdstock, Volker Brenner, Marcus Harrison, Colin P Smith

**Affiliations:** 1 Microbial Sciences Division, Faculty of Health and Medical Sciences, University of Surrey, Guildford, GU2 7XH, UK; 2 Oxford Gene Technology Ltd, Begbroke Business Park, Sandy Lane, Yarnton, Oxford OX5 1PF, UK; 3 Current address: Institute of Immunology, Biomedical Sciences Research Centre "Alexander Fleming", Athens 16672, Greece

## Abstract

**Background:**

DNA microarrays are a key resource for global analysis of genome content, gene expression and the distribution of transcription factor binding sites. We describe the development and application of versatile high density ink-jet *in situ*-synthesized DNA arrays for the G+C rich bacterium *Streptomyces coelicolor*. High G+C content DNA probes often perform poorly on arrays, yielding either weak hybridization or non-specific signals. Thus, more than one million 60-mer oligonucleotide probes were experimentally tested for sensitivity and specificity to enable selection of optimal probe sets for the genome microarrays. The heat-shock HspR regulatory system of *S. coelicolor*, a well-characterized repressor with a small number of known targets, was exploited to test and validate the arrays for use in global chromatin immunoprecipitation-on-chip (ChIP-chip) and gene expression analysis.

**Results:**

In addition to confirming *dnaK*, *clpB *and *lon *as *in vivo *targets of HspR, it was revealed, using a novel ChIP-chip data clustering method, that HspR also apparently interacts with ribosomal RNA (*rrnD *operon) and specific transfer RNA genes (the tRNA^Gln^/tRNA^Glu ^cluster). It is suggested that enhanced synthesis of Glu-tRNA^Glu ^may reflect increased demand for tetrapyrrole biosynthesis following heat-shock. Moreover, it was found that heat-shock-induced genes are significantly enriched for Gln/Glu codons relative to the whole genome, a finding that would be consistent with HspR-mediated control of the tRNA species.

**Conclusions:**

This study suggests that HspR fulfils a broader, unprecedented role in adaptation to stresses than previously recognized - influencing expression of key components of the translational apparatus in addition to molecular chaperone and protease-encoding genes. It is envisaged that these experimentally optimized arrays will provide a key resource for systems level studies of *Streptomyces *biology.

## Abstract 2

Development of high-density microarrays for global analysis of gene expression and transcription factor binding in Streptomyces coelicolor  suggests a novel role for HspR in stress adaptation.

## Background

Streptomycetes represent an unusual and complex bacterial genus. They display a mycelial 'multicellular' life cycle that culminates in sporulation [1] and possess remarkable metabolic diversity, both in their ability to catabolise complex substrates and in their prodigious capacity to produce chemically diverse 'secondary' metabolites, including the majority of naturally occurring antibiotics and other bioactive compounds used in medicine [2,3]. These characteristics form the major justification for basic studies of streptomycete biology. Since the completion of the genome sequence of the principal model streptomycete, *Streptomyces coelicolor *A3(2) [4], numerous systems-level studies have been initiated, encompassing transcriptomic/proteomic approaches and genome scale metabolic network construction [5-8].

To date, *Streptomyces *DNA microarray-based studies have been restricted largely to the use of spotted PCR products or pre-synthesized long oligonucleotides, with a single probe representing each gene [9]. Such arrays are not generally suitable for genome wide chromatin immunoprecipitation-on-chip (ChIP-on-chip) analysis of transcription factor binding sites [10]. The ChIP-on-chip technique has become an essential tool for system wide analysis of biological systems (for example, [11-15]) since it provides a comprehensive assessment of the direct targets, *in vivo*, of the transcription factor/DNA-binding protein under investigation; this is a pre-requisite for reconstructing cellular transcription regulatory networks. Here we report the development of ink-jet *in situ *synthesized (IJISS) DNA arrays for ChIP-on-chip analysis of *S. coelicolor*.

Streptomycetes are unusual in possessing genomes of very high G+C content. The *S. coelicolor *genome is 72.4% G+C and individual coding sequences often exceed 80% G+C. This extreme base composition compromises the design of suitable probes for array-based detection of complementary nucleic acid sequences because G+C-rich probes often hybridize poorly with targets or they display a lack of specificity. Consequently, in this study we adopted an experimental approach to test a large collection of arrayed probes for sensitivity and specificity prior to selecting a subset for the final genome arrays. The objective was to produce a versatile experimentally optimized array that could be used for both genome-wide ChIP-on-chip analysis and global gene expression profiling.

The HspR heat-shock regulatory system of *S. coelicolor *[6] was exploited to test and validate the sensitivity and specificity of the IJISS arrays. HspR was selected because it represents a well-characterized repressor with a small number of known targets. Streptomycetes have adopted diverse strategies to rapidly adjust to sudden changes in the environment, for example, from heat stress or other physico-chemical and physiological stresses. As in all living organisms, they induce expression of many genes in response to heat stress, including the well characterized and universally conserved members of the *hsp70 *(*dnaK*) and *hsp60 *(*groEL*) gene families (see [16-18] for reviews). In *Streptomyces *and most Gram-positive and Gram-negative bacteria the heat shock stimulon is under the control of negative transcriptional regulators [19], unlike *Escherichia coli *where the heat shock stimulon is under the positive regulation of the alternative sigma factors σ^32 ^and σ^24 ^[20,21]. The heat shock stimulon mostly comprises two major classes of genes encoding, respectively, molecular chaperones and proteases that are induced under conditions that cause protein misfolding/denaturation in order to maintain protein quality control, or eliminate protein aggregates or badly damaged proteins that would otherwise have a deleterious effect on cell survival.

Three negative transcriptional regulators have been characterized in *Streptomyces *species: HrcA, controlling the *groES*/*EL1 *operon and *groEL2 *(for a review, see [22]); RheA controlling *hsp18 *in *Staphylococcus albus *[23,24]; and HspR controlling the *dnaK *operon, *clpB *molecular chaperone and *lon *protease-encoding genes [25-27].

The HspR repressor has since been identified in some other bacterial systems: *Mycobacterium tuberculosis*, where it controls the expression of the *hsp70 *operon, *clpB *and *acr2 *genes [28]; and *Corynebacterium glutamicum *[29], where it controls the *clpP1*/*P2 *operon together with two other regulators, ClgR and σ^H^. Furthermore, the HspR system has been reported in other bacteria not belonging to the Actinomycetales family, such as the Gram-negative *Helicobacter pylori*, where HspR functions in conjunction with HrcA to regulate the *groES*/*EL *and *hrcA-dnaK-grpE *operons [30-32], *Deinococcus radiodurans*, where HspR controls two novel members of the regulon (*hsp20 *and *ftsH*) in addition to known members such as *dnaK, dnaJ, grpE, lonB* and *clpB *[33], *Bifidobacterium breve *[34] and *Campylobacter jejuni *[35].

In the present study we have optimized methods for chromatin immunoprecipitation and have produced optimized high density arrays for ChIP-on-chip analysis of *S. coelicolor*. Here we exploit this technology (to our knowledge applied for the first time with *Streptomyces*) to redefine the HspR regulon of *S. coelicolor*. The microarray design allows gene expression data to be superimposed for the same probes, enabling discrimination between indirect effects of either over-expressing or disrupting a regulator gene from the direct effect resulting from the *in vivo *binding of the respective regulator to its target genes. In addition to confirming *dnaK*, *clpB *and *lon *as *in vivo *targets of HspR, the ChIP-on-chip studies reported here indicate that HspR also has a role in regulation of expression of ribosomal RNA and specific transfer RNA genes, for incorporation of Gln and Glu, the latter potentially linked with tetrapyrrole biosynthesis. This suggests that HspR fulfils a broader role in adaptation to stresses, such as heat-shock, than was previously recognized - influencing expression of key components of the translational apparatus in addition to major molecular chaperone and protease-encoding genes. It is envisaged that these IJISS arrays will find wide application in systems level studies of *Streptomyces *biology.

## Results and discussion

### DNA microarray design

**Figure 1:**
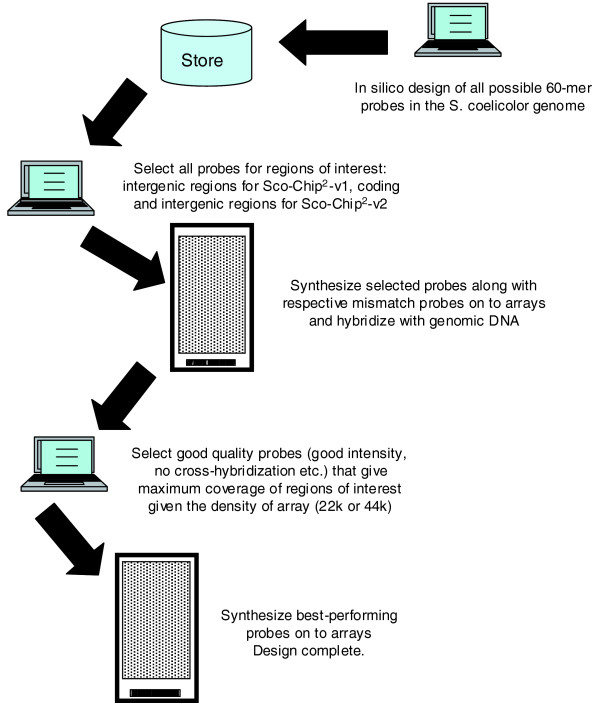
**Overview of array design strategy**.

Two different *S. coelicolor *IJISS DNA microarrays were designed, featuring, respectively, 22,000 (Sco-Chip^2^-v1) and 44,000 (Sco-Chip^2^-v2) 60-mer oligonucleotide probes. In each case the same experimental optimization approach was used (Figure 1) where a large set (approximately 1 million) of 60-mer probes were printed in parallel with corresponding probes that had a 3-nucleotide mismatch. Cyanine-3 (Cy3) and Cyanine-5 (Cy5)-labeled *S. coelicolor *genomic DNA was hybridized against the test arrays and the probe performance was scored using the following equations. Firstly the Cy3 and Cy5 background-subtracted signals, designated 'g' and 'r', respectively, obtained by feature extraction of the arrays using the Agilent feature extraction software (Version 9.1.3.1) were entered into the following formula:

A = (gMM/gPM + rMM/rPM)/2

where the signal from the perfectly matched probe is designated 'PM' while that from the corresponding mismatched probe is designated 'MM'. For values of A greater than 1, A was set to 1 before entering it into the second equation:

R = [1 - arctan (A × π/2)] × [1 - exp(- (gPM + rPM)/2000)]

The resulting R-value was used to rank all tested probes. The higher the value, the better the probe performance. This method of ranking probes was developed within Oxford Gene Technology Ltd and has been applied to various prokaryotic organisms for empirical microarray probe design.

Sets of probes within a defined region, either gene or intergenic, were ranked. All probes were considered relative to each other without applying thresholds and the desired density of probe coverage was achieved by selecting top-ranked probes where possible. Performing the above experimental optimization approach is, in our opinion, a necessary step, given that approximately 40% of the *in silico *designed probes failed quality control. Sufficient probe coverage was obtained using fewer than 5% of probes ranked below the median value of the ranking distribution. The remaining 95% of the optimized probe set were picked from probes performing above average with a strong bias for very well performing probes. For both array formats the probes were deposited at random positions on the slide surfaces to minimize the risk of any position-specific artifacts.

### Sco-Chip^2^-v1 array

All possible 60-mer probes for all targets (both coding and non-coding sequences) in the *S*. *coelicolor *genome (based on the *S*. *coelicolor *A3(2) [EMBL:AL645882.2]) were designed. For this version of the array all non-coding sequences upstream of protein-encoding genes were selected (sequences where transcription factors are most likely to bind) and multiple 60-mer probes targeting those regions were selected from the 'all possible probes' set. Following this initial selection, a total of 84,268 probes were experimentally tested and the best performing 21,064 probes that represented all upstream intergenic regions (an average of 3 approximately 110 bp spaced probes to each upstream site) in the genome were synthesized on the array. As this array design was developed specifically for ChIP-on-chip experiments, all probe sequences corresponded to one strand only (that in [EMBL:AL645882.2]) since the particular DNA strand was unimportant. (Note that intergenic regions flanked by transcription terminators for convergently transcribed genes were not selected for this array.)

### Sco-Chip^2^-v2 array

From the 'all possible probes' set (see above), 964,820 60-mer probes were selected and printed to target all coding and non-coding sequences with minimal distance between the probes and maximal coverage of the genome. Following experimental validation of probe signal and specificity, 43,798 of the best performing probes were selected to give broad coverage. Probes within protein coding sequences corresponded to the mRNA strand for (cDNA-based) detection of gene expression. For intergenic regions the probe sequences corresponded to one strand only (that in [EMBL:AL645882.2]). The average spacing of probes in the genome was approximately 135 bp.

### Genome-wide identification of *in vivo *HspR binding sites

The experimentally optimized Sco-Chip^2^-v1 and Sco-Chip^2^-v2 arrays were used consecutively to identify *in vivo *targets of HspR. The latter array was designed to also enable quantification of gene expression. In order to validate the sensitivity and specificity of these arrays, we chose the well-studied transcriptional repressor HspR, which was previously known to bind to only three promoter regions in the genome of *S. coelicolor*: upstream of the *dnaK *operon; the protease-encoding gene *lon*; and the *clpB *gene, which is transcribed in an operon with *SCO3660*. These results were based on transcriptome analysis of an *hspR *disruption mutant and complementary *in silico *genome wide searches for HspR binding sites [6].

**Figure 2:**
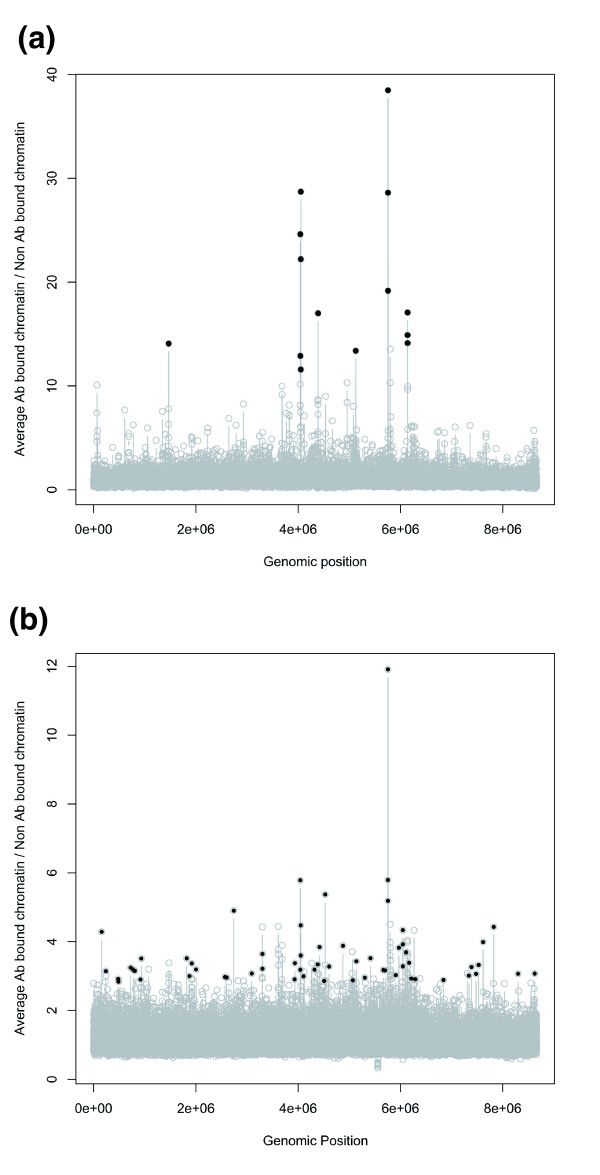
**HspR-mediated enrichment of array probes across the *S. coelicolor *genome**. Black dots indicate probes identified as being significantly enriched (see Materials and methods). Note that there are multiple probes for each gene/intergenic region. **(a) **Probes identified with array Sco-Chip^2^-v1. The list of significant probes is given in Additional data file 12. **(b) **Probes identified with array Sco-Chip^2^-v2 (listed in Additional data file 13).

**Figure 3:**
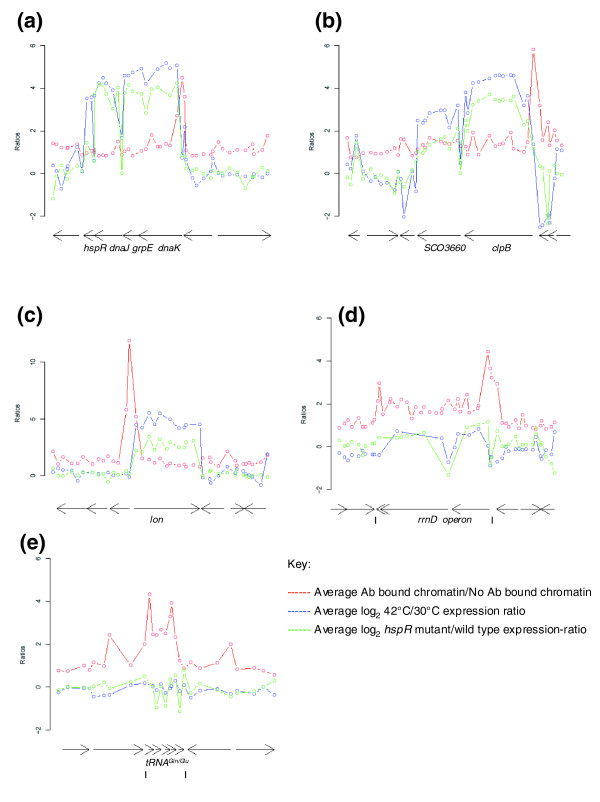
**Probe signals across significantly scoring HspR target regions using the Sco-Chip^2^-v2 array**. **(a) **the *dnaK *operon, **(b) **the *clpB *(SCO3661) operon, **(c) **the *lon *gene (*SCO5285*), **(d) **the *rrnD *ribosomal RNA operon and **(e) **the tRNA^Gln/Glu ^cluster. The anti-HspR-enriched probes are plotted on a linear scale (red), the heat-shock expression ratio at 42°C versus 30°C is plotted in log_2 _scale (blue), and the expression ratio of the *hspR *disruption mutant versus wild-type is plotted in log_2 _scale (green). Open circles indicate the start co-ordinate (relative to genome sequence) of each probe that passed quality control filtering. The genetic organization of each region is indicated below the plot; each arrow represents a coding sequence or stable RNA gene as defined in [EMBL:AL645882.2].

For the ChIP-on-chip experiments, samples of *S. coelicolor *cultures at early stationary phase were treated with formaldehyde and subjected to immunoprecipitation (IP) as described in Materials and methods. For these experiments, *S. coelicolor *was cultivated under non-heat-shock conditions in a rich liquid medium containing 10.3% sucrose to support dispersed growth of the mycelium and provide sufficient biomass for the ChIP protocol; this was to maximize formaldehyde penetration and to determine the genomic distribution of HspR under non-stressed conditions (the 'resting' state). Following the IP reaction, the DNA was recovered, labeled with Cy3-dCTP and then co-hybridized onto the Sco-Chip^2^-v1 and Sco-Chip^2^-v2 arrays together with the Cy5-dCTP-labeled total chromatin as reference (Sco-Chip^2^-v1) or with Cy5-dCTP labeled mock 'no-antibody' IP chromatin (Sco-Chip^2^-v2) (see Materials and methods). The results presented in Figures 2 and 3 (and Additional data file 3) represent the average of two biological replicates. They confirm that HspR does bind, *in vivo*, to the *dnaK*, *clpB *and *lon *promoter regions and, importantly, have served to identify additional putative HspR targets. The statistical approaches used to score probe enrichment ratios (gene targets) as significant differed between the two array formats because in Sco-Chip^2^-v1 the probes were focused only on promoter regions while in Sco-Chip^2^-v2 the probes were relatively evenly spaced across the genome (see Materials and methods). The targets scored as significant using Sco-Chip^2^-v1 were *dnaK*, *clpB*, *lon *and *SCO5639 *and those on Sco-Chip^2^-v2 were *dnaK*, *clpB*, *lon *and probe sequences between *SCO3019*-*SCO3020 *and *SCO5549*-*SCO5550*, corresponding, respectively, to the promoter region of the *rrnD *ribosomal RNA operon and a five-tRNA cluster encoding tRNA^Gln ^and tRNA^Glu ^species; if the cut-off threshold was slightly relaxed for the Sco-Chip^2^-v2 data, then *SCO5639 *was also identified.

The respective nucleotide sequences of the new putative stable RNA targets of HspR had been excluded from Sco-Chip^2^-v1 because they are non-protein-coding and are positioned between convergently transcribed genes. The discovery that HspR may regulate specific tRNA and rRNA genes is unprecedented and suggests a more global role for HspR in the stress response of *Streptomyces*. The HspR-specific probe enrichments in the previously known and the new putative HspR targets are shown in Figure 3 (and Additional data file 3).

### The heat-shock stimulon of *S. coelicolor*

The versatility of the Sco-Chip^2^-v2 design allowed us to detect gene expression using the same probe set. Thus, in Figure 3 the expression data from two pairs of comparisons are also superimposed on the ChIP enrichment data: the ratio of expression from *hspR *disruption mutants relative to the wild-type strain; and the ratio of expression from cultures heat-shocked at 42°C relative to non-heat-shocked control cultures. It is noted that the observed reduction of relative transcript levels of operonic genes more distal from the operon promoter (as in the *dnaK *operon; Figure 3a) is consistent with general observations of polarity of expression of *Streptomyces *operons [36].

The gene expression studies were conducted using RNA samples from strains cultivated on supplemented minimal medium agar plates, rather than rich liquid medium. This was for several reasons. First, the magnitude of the heat-shock response is relatively lower and less reproducible in heat-shocked mycelium cultivated in the rich YEME+10.3% sucrose liquid medium, compared with the heat-shock response of the surface-grown minimal medium cultures. Second, the *hspR *disruption mutants used in this study are unstable because *hspR *is an essential gene [6,27]. The disruption of *hspR *is via a single integration event of a non-replicating plasmid and there is a strong selective pressure for its excision. Thus, in liquid culture the mycelium in which the disruption plasmid has excised outgrows the mutant mycelium, which is at a growth disadvantage, leading to a dominant wild-type revertant phenotype. In surface-grown cultures this reversion is markedly attenuated, where a low frequency of reversion maintains viability. Third, the RNA samples used here for comparison with the ChIP data have been extensively validated by other methods [6,27]. The above experiment allowed, for the first time, a comprehensive identification of the heat-shock stimulon of *S. coelicolor *at the transcriptome level, where rank products analysis revealed 119 up-regulated genes (based on a probability of false prediction (pfp) threshold of <0.15 (see Materials and methods)) as a result of heat-shock (Additional data file 4). The use of such thresholds has been reported elsewhere [7,37].

Two genes on the heat-shock list with relatively high pfp values (*SCO3202*, pfp = 0.12; *SCO4157*, pfp = 0.13) were selected for independent validation by quantitative real time PCR (qPCR) to confirm true heat-shock induction (Additional data file 9); furthermore, *SCO3660*, a known member of the HspR regulon [6], had a pfp value of 0.12. This justified the use of the pfp threshold adopted here. The significantly up-regulated heat shock genes include all members of the *dnaK *operon, *clpB*, *lon *and the chaperonin-encoding *groES*-*groEL1 *operon and *groEL2*. Two more protease-encoding genes are also present in the heat-shock list: *SCO4157*, encoding the homologue of *E. coli *HtrA, a serine protease involved in degradation of periplasmic misfolded proteins, and *SCO6515*. Notably, eight oxidoreductase-encoding genes are present, some of them being strongly up-regulated by heat-shock and transcribed in an operon (*SCO1131*-*SCO1134*). The operon encoding different subunits of the nitrate reductase (*SCO0216*-*SCO0219*) and the nitrite/nitrate transporter-encoding gene *SCO0213 *are also heat-inducible together with the principal 'gas vesicle protein'-encoding operon (*SCO6499*-*SCO6508*) [38], the cytochrome oxidase-encoding genes *SCO3945*-*SCO3946 *and two genes of *sigE *operon (*sigE *(*SCO3356*) and the lipoprotein-encoding gene *cseA *(*SCO3357*)) [39]. It is of interest that more than 10% of the heat-shock-induced genes (14) encode transcriptional regulators and included *SCO0174 *(the most induced), five sigma factors (HrdD (*SCO3202*), SigB (*SCO0600*), SigE (*SCO3356*), SigL (*SCO7278*) and SigM (*SCO7314*)) and an anti-sigma factor antagonist (*SCO7325*). A separate, complementary analysis of the heat-shock response in wild-type *S. coelicolor *cultivated under identical conditions in YEME to those used for the ChIP-on-chip studies demonstrated that most of the above 119 heat-shock induced genes (102/119) were also heat-induced in the YEME medium (Additional data file 11); however, the level of induction of the well-known molecular chaperone-encoding genes was attenuated relative to the surface grown SMMS cultures. It is interesting to note that 16 of the 17 genes not heat-induced in the YEME cultures are clustered in a discrete region at the left end of the chromosome between *SCO162 *and *SCO219*; this may reflect the differences in the widely different nutritional compositions of the two growth media and these genes may require additional transcription factors for their induction.

The list of 55 genes significantly up-regulated in an *hspR *disruption mutant relative to the wild-type is presented in Additional data file 5 (cut-off pfp < 0.15). It includes all previously known members of the HspR regulon and other notable genes that, on the basis of the ChIP-on-chip analysis, are not considered to be directly controlled by HspR; their induction could be a consequence of the up-regulation of molecular chaperone or protease-encoding gene expression in the HspR mutant. Genes for five putative transcriptional regulators are represented in the list.

### New putative targets of HspR

The sensitivity of the IJISS arrays was deduced to be high since all previously known HspR targets were identified on both array designs. *SCO5639 *was not identified as belonging to the HspR regulon in a previous study [6]. *SCO5639 *encodes a hypothetical protein of 176 amino acids in length and, unusually for streptomycete genes, has a low G+C content (approximately 53%) and is flanked by genes also of low G+C content: *SCO5638 *(55% G+C), which encodes an integral membrane protein, and *SCO5640 *(54% G+C), which encodes a hypothetical protein. Moreover, the adjacent gene, *SCO5641*, encodes a putative transposase, suggesting that *SCO5639 *could have been laterally acquired recently. Pfam [40] searches of the deduced amino acid sequence of SCO5639 returned the 'domain of unknown function' DUF1863, corresponding to a domain that adopts the 'flavodoxin fold' with "a probable role in signal transduction as a phosphorylation-independent conformational switch protein". Other proteins that contain this domain (37 known in total, including another actinomycete, *Corynebacterium efficiens*) are also uncharacterized. Similarly, blastp [41,42] results identified further hypotheticals (at E-value < 1^e-10^) and found a similarity, albeit low (35% identity), to the phosphorylation site of the calcineurin temperature suppressor (*Cts1*) of *Cryptococcus neoformans *(a yeast), which is responsible for restoring growth of calcineurin mutant strains at 37°C among other functions such as cell separation and hyphal elongation [43]. This link with temperature would be consistent with *SCO5639 *being a target of HspR.

#### The HspR binding motif

**Figure 4:**
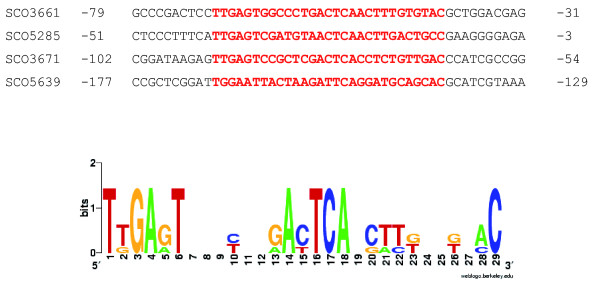
**Consensus operator sequence for HspR**. The nucleotide sequence was determined by the alignment of the upstream regions of HspR targets identified by Sco-Chip^2^-v1 (see Materials and methods). The sequences are displayed above the consensus plot; numbering of nucleotides is relative to the predicted start codon of each gene. In the graphical representation of the consensus sequence the height of each nucleotide indicates the level of conservation [76,77].

In previous work the minimal consensus operator for HspR binding, generated by the alignment of upstream sequences of *clpB*, *lon *and *dnaK*, was documented as 5'-TTGAGYNNNNNNNACTCAA [6]. A MEME (Maximum Em for Motif Elicitation) alignment (see Materials and methods) of the upstream sequences of these three genes and *SCO5639 *produced a modified consensus sequence of 5'-TKGARTNNNYNNRAYTCA (Figure 4). This new consensus sequence was used to search the *S*. *coelicolor *genome using RSAT [44,45] with default settings; five matches were found, *SCO4410 *and the above four genes.

#### *In vitro *analysis of new HspR targets

**Figure 5:**
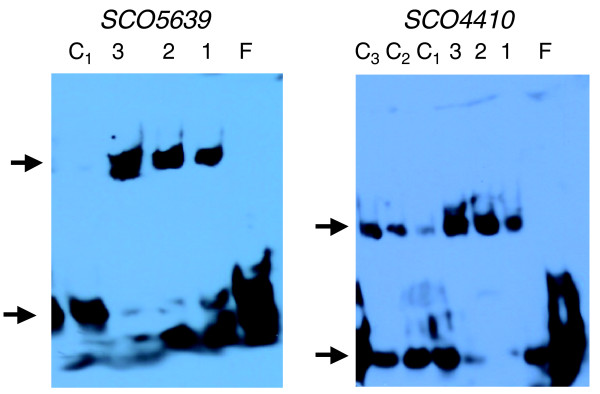
**Gel-shift assays of putative new HspR targets: *SCO5639 *and *SCO4410***. HspR-binding at the *SCO5639 *and *SCO4410 *promoter regions. Oligonucleotide pairs are detailed in Materials and methods. Protein extract from *E. coli *over-expressing *hspR *was incubated with 200 fmol biotinylated DNA fragment without competitor DNA (lanes 1-3) with, respectively, 4, 6 and 12 μg cell extract. In the lane marked 'C_1_' in the *SCO5639 *gel shift, 4 μg cell extract and 200-fold molar excess of specific competitor DNA were loaded. In lanes C_1_-C_3 _in the *SCO4410 *gel shift, 4, 6 and 12 μg cell extract were loaded, respectively, together with 200-fold molar excess of specific competitor DNA. Lane F shows unbound DNA (no added protein). Arrows indicate positions of bound (upper arrows) and unbound double-stranded DNA target.

Gel shift assays were conducted using DNA sequences upstream of the *dnaK *operon as a positive control [26] (data not shown), *SCO5639 *and *SCO4410*. The results indicate that HspR binds *in vitro *to the putative HspR motifs of *SCO5639 *and *SCO4410 *(Figure 5). However, a convincing gel-shift was only obtained for *SCO5639 *and *SCO4410 *with the HspR-containing *E. coli *cell extracts and not with the purified, refolded, HspR, suggesting that additional factors might be required for an efficient binding/stabilization of HspR at the *SCO5639 *and *SCO4410 *promoters; such factors would need to have a counterpart in *E. coli *to explain these results. An alternative explanation could be that the HspR-DnaK complex in the *SCO5639 *and *SCO4410 *promoter regions is weaker and that this binding is not necessarily responsible for modulating heat-shock regulation of these genes. Expression of these two genes following heat-shock and in a *hspR *disruption mutant was assessed by qPCR (Additional data files 9 and 10). *SCO5639 *was only modestly heat-induced (by 23%) but, importantly, it was up-regulated approximately fivefold in a *hspR *disruption mutant. It is possible that *SCO5639 *is co-regulated by other factors that are not influenced directly by heat. *SCO4410*, a very poorly expressed gene, was induced approximately 3-fold by heat-shock and it was up-regulated approximately 15-fold in a *hspR *disruption mutant. On the basis of these results, *SCO4410 *and *SCO5639 *are considered to be genuine targets of HspR. The *SCO4410 *gene, which is predicted to encode an anti-anti-sigma factor was not identified in the ChIP-on-chip experiments. It is known that false negatives occur in such studies and they are considered to arise due to sequestration of the transcription factor in nucleoprotein complexes, rendering them inaccessible to the specific test antibody used for the IP reaction; it should be noted that one of the best studied protein-DNA complexes, the CRP-*lac *promoter complex of *E. coli*, was not identified in ChIP-on-chip analysis of CRP binding in *E. coli *[12].

#### Stable RNA genes as putative targets for HspR

The observation that HspR appears to bind to the promoter region of the *rrnD *operon and to multiple sequences within the five-tRNA^Gln/Glu ^cluster is unprecedented. Other than the previously known HspR targets, these were the only two regions identified as statistically significant by the data clustering method reported in this study for the Sco-Chip^2^-v2-derived data. Previous studies would not have identified stable RNA genes as potential targets because representative probes had not been printed on the arrays. Furthermore, the typical 'transcription factor binding site' consensus sequence identification technique is based on searching the upstream regions of protein-encoding genes and/or a set threshold is applied *in silico*, which may not be able to simulate the true *in vivo *binding that occurs. Indeed, the *in silico *analysis carried out in this study (discussed in Materials and methods), revealed only partial recognition of the defined HspR consensus (Additional data file 6) whilst the *in vivo *data (ChIP-on-chip enrichment ratios) indicate HspR binding. Thus, it is likely that other transcription factors also bind to these regions, or that other DNA sequences facilitate HspR-binding, and it is possible that such factors could positively influence the binding of HspR, relieving its dependence on a substantial consensus sequence match. In this context it is notable that the most highly enriched probes flank both the beginnings and ends of the *rrnD *operon and five-tRNA cluster (Figure 3d,e, respectively); it is conceivable that HspR forms a looped complex at both of these stable RNA-encoding regions. A MEME analysis (see Materials and methods) revealed a non-palindromic motif shared between the HspR targets identified with the Sco-Chip^2^-v2 array (Additional data file 7); the biological significance of this motif is not clear.

HspR regulates the DnaK chaperone machine, a system that plays an important role in the cotranslational folding of proteins [46] in addition to assisting folding of unfolded or partially unfolded mature polypeptides. It could be rationalized that HspR inactivation also facilitates expression of rRNA and tRNAs following heat-shock, or other stresses, as part of the transient adaptive response to environmental stresses. From the gene expression analysis (Figure 3; Additional data files 14 and 15) there is a detectable enhancement (albeit small) of *rrnD *and tRNA^Gln/Glu ^transcript levels in heat-shocked cultures and in an *hspR *disruption mutant; a high over-representation would not be expected because these particular stable RNA genes are highly expressed under normal growth conditions (data not shown) and a transient (approximately 15 minute) induction of one of the rRNA operons would not have a major impact on the large pre-existing pool of these stable species within the cytoplasm. From averaging of signals from the multiple probes in this region, the increase in the *rrnD *16S rRNA transcript level was ≥ 10% following heat-shock and ≥ 20% in an *hspR *disruption mutant. We suggest that HspR-mediated control of *rrnD *transcription facilitates the maintenance of rRNA transcription following heat-shock. There are precedents for heat-stimulated transcription of rRNA operons in both *Streptomyces *and *E. coli*. León and Mellado demonstrated partial heat-shock stimulation of some rRNA promoters in the closely related species *S. lividans *[47]. In *E. coli *the heat-shock sigma factor σ^32 ^was shown to direct transcription of the *rrnB *P1 promoter and the authors suggest that σ^32^-directed transcription of rRNA promoters might play a role in ribosome synthesis at high temperatures [48]. There are also reports of developmental regulation of rRNA and ribosomal protein synthesis in *S. coelicolor *[49,50]. The upstream region of *rrnD *of *S. coelicolor *displays significant differences from that of other rRNA promoters in this genome (*S. coelicolor *contains six *rrn *operons). In this context it is of relevance that a recent study suggests that the p3 and p4 promoters of *rrnD *are differentially regulated by additional (as yet unidentified) factors [51]. It is tempting to speculate that HspR-mediated induction of *rrnD *transcription may result in the production of a subset of ribosomes with a specific role in translation of stress-responsive proteins.

Transient stimulation of transcription of the tRNA^Gln/Glu ^cluster may lead to an enhancement in the cellular level of uncharged tRNAs - particularly since Gln-tRNA^Gln ^formation requires transamidation of Glu-tRNA^Gln ^[52]. In this context it might be relevant that the tRNA^Gln/Glu ^cluster encodes the only two tRNA^Gln ^species in *S. coelicolor *and the transient accumulation of uncharged tRNAs is known to be a major trigger for the stringent response [53].

Most organisms contain only one Glu-tRNA^Glu ^species [54]. The tRNA^Gln/Glu ^cluster identified in this study encodes three Glu-tRNA^Glu ^species (recognizing the GAG codon); one other Glu-tRNA^Glu ^gene is encoded elsewhere in the *S. coelicolor *genome and recognizes the GAA codon, which is a very rarely used streptomycete codon. Glu-tRNA^Glu ^has two major roles in the cell. In addition to its role in protein synthesis, Glu-tRNA^Glu ^is a substrate in the first step of tetrapyrrole biosynthesis, to produce heme, for example [54,55]. Inspection of the predicted protein products from the *S. coelicolor *genome indicates that this is the only available route for tetrapyrrole biosynthesis in this organism. It is possible, therefore, that there is an enhanced requirement for tetrapyrrole production following heat-shock (and concomitant oxidative stress), to provide heme, for example, for cytochrome biosynthesis and for catalase and superoxide dismutase production and this could be achieved by HspR-mediated regulation of Glu-tRNA^Glu ^expression.

An additional possible explanation for enhanced expression of this sub-set of tRNAs could be that there is a higher transient demand for Gln and Glu in protein synthesis immediately following heat-shock. Transcript levels of two of the five tRNA species was enhanced approximately 10% in *hspR *disruptants (Figure 3; Additional data file 15). Indeed, the Gln/Glu frequency (the percentage of Gln/Glu codons in a codon set) in the heat-shock up-regulated gene set (Additional data file 4) is higher than that obtained for the entire genome (9.76% versus 8.32%). To estimate the significance of this finding, 10,000 random subsets of genes from the entire genome, of the same size as the up-regulated gene list, were created and their Gln/Glu frequency was calculated. It was found (through the use of the Z-score) that the heat-shock up-regulated gene set had an enhanced Gln/Glu codon frequency compared to any of the random sets, yielding a significance *p*-value of < 1.06 × 10^-7^. It is concluded that there is statistically significant enrichment of Gln/Glu codons in the heat-shock up-regulated genes and we speculate that HspR mediates transient stimulation of expression of the relevant tRNAs. Although the biological significance of this finding is not clear, it may be relevant that Glu (and Lys) tend to be over-represented in thermostable proteins [56]. The difference in amino acid composition of the heat-shock genes relative to all genes, for all amino acids and amino acid pairs, is given in Additional data file 8.

## Conclusion

High density IJISS DNA arrays have been developed for global analysis of *Streptomyces *gene expression and transcription factor binding. The HspR regulatory system of *S. coelicolor *was exploited to validate their sensitivity and specificity. New insights were gained into the possible role of HspR in regulation of cellular physiology - encompassing stable RNA synthesis in addition to molecular chaperone and protease production. It is envisaged that these arrays will find widespread use in systems level analysis of *Streptomyces coelicolor *biology.

## Materials and methods

### *Streptomyces *strains and culture conditions

For the ChIP-on-chip studies the prototrophic *S. coelicolor *strain MT1110, a SCP1^- ^SCP2^- ^derivative of the wild-type strain, John Innes Stock Number 1147 [57], was cultivated in YEME liquid medium plus 10% sucrose at 30°C in a rotary shaking incubator. For the gene expression studies the previously reported two independent *hspR *disruption mutants, MT1151 and MT1153, were used together with the two independent, otherwise isogenic, *hspR*^+ ^integrants, MT1152 and MT1154 [58]. The heat-shock conditions were as reported previously [26].

### Chromatin immunoprecipitation

In order to obtain *Streptomyces *chromatin of high quality, it was found that rapid, low temperature, physical disruption of the mycelium constituted a more reproducible method than the conventional lysozyme treatment methods. *S. coelicolor *MT1110 was cultivated at 30°C in 50 ml YEME liquid medium in 250 ml flasks with springs (supplemented with 10% sucrose, glycine and MgCl_2 _as specified in [58] up to early stationary phase (OD_450 _approximately 2.0). Cultures were divided into 20 ml aliquots and formaldehyde treated (final concentration, 1%) for 10 minutes at 30°C in order to *in vivo *crosslink proteins to DNA; glycine (final concentration of 0.5 M) was added to quench the formaldehyde and the culture was incubated for a further 5 minutes at 30°C. Mycelium was harvested by centrifugation, frozen in liquid nitrogen and then transferred to a 7 ml PTFE shaking flask with cap (which was also immersed in liquid nitrogen to cool it down). Mycelium was disrupted in a Mikrodismembrator U mechanical device (Sartorius Stedim Biotech, Epsom, Surrey, UK) for 2 × 1 minute at 2,000 rpm with one 10 mm diameter chromium steel grinding ball and contents of one tube of lysing matrix B (Q-BIOgene, Cambridge, UK). Chromatin processing and IP were based on previous methods [59,60] with additional modifications. The pulverized mycelium was transferred to a tube containing 1 ml lysis buffer (10 mM Tris-HCl, pH 8, 20% sucrose, 50 mM NaCl, 10 mM EDTA, Protease Inhibitor Cocktail (Roche, Burgess Hill, West Sussex, UK); one tablet per 10 ml); 3 ml IP buffer (50 mM Tris-HCl, pH 8, 150 mM NaCl, 0.5% Triton X-100, plus Protease Inhibitor Cocktail) was added and the chromatin was sheared by sonication (Sonics VibraCell VCX130, CH-1217 Meyrin/Satigny, Switzerland) on ice. One 2 ml aliquot was sonicated 2 × 20 s power ON at 50% power, 40 s power OFF and the other 2 ml chromatin sample was sonicated 4 × 20 s power ON at 50% power, 40 s power OFF, to obtain the optimal DNA size range of 0.5-1.0 kb. Cell lysates were cleared by centrifugation at 12,000 rpm for 25 minutes. To assess chromatin quality, aliquots of the chromatin (70 μl) were treated with proteinase K (100 μg, Roche) for 2 h and the DNA-protein complexes were de-crosslinked at 65°C for 6 h; 5 μl aliquots were subjected to electrophoresis and the chromatin fraction(s) with optimal size range were subjected to IP either with specific antibody or with no antibody (mock IP) as control. The IgG fraction containing anti-HspR polyclonal antibodies [61] and the fraction from pre-immune serum from the same rabbit used for immunization were purified through Nab Protein A spin columns (Pierce, ThermoFisher Scientific, Cramlington, Northumberland, UK). Chromatin IP was carried out with 100 μl specific antibody added to 800 μl of chromatin overnight at 4°C on a rotating wheel at 12 rpm; 80 μl of either sepharose protein A (Sigma, Gillingham, Dorset, UK) or Ultralink immobilized Protein A/G beads (Pierce; previously washed twice in phosphate-buffered saline (PBS), once in PBS containing 5 μg/ml bovine serum albumin and resuspended in one half bead volume of PBS containing a protease inhibitor cocktail) were added to the immunoprecipitated chromatin and incubated for a further 3-4 h at 4°C on a rotating wheel at 12 rpm. The DNA-protein complexes bound to the beads were pelleted at 3,300 rpm for 1 minute and washed four times by resuspension in 1 ml ice cold IP buffer (wash 1), IP buffer plus salt (wash 2, as wash 1 but with 500 mM NaCl), wash buffer (wash 3, 10 mM Tris pH 8, 250 mM LiCl, 1 mM EDTA, 0.5% nonidet P-40 and 0.5% Na deoxycholate) and TE pH 7.6 (wash 4), with incubation at 4°C in a rotating wheel for 15 minutes and centrifugation at 3,300 rpm for 1 minute. After the first wash the protein A/G bound DNA protein complexes were transferred to a non-stick microfuge tube.

Immunoprecipitated complexes were eluted overnight at 55°C in Tris-EDTA, pH 7.6 (TE), 1% SDS, 100 μg Proteinase K (Roche) in 240 μl volume. A 170 μl aliquot of input chromatin (not subjected to IP) or mock-IP chromatin was incubated in parallel under the same conditions with 240 μl of elution buffer. Crosslinks were dissociated at 65°C for 30 minutes followed by centrifugation at 3,300 rpm for 1 minute. The protein A/G beads were washed in 50 μl TE and the supernatants were pooled. The immunoprecipitated and input chromatin/mock-IP samples were extracted twice with phenol/chloroform/isoamyl alcohol (25:24:1) pH 8, then once with chloroform and the DNA was ethanol precipitated in the presence of 20 μg glycogen as carrier, resuspended in 20 μl ultrapure water and quantified with a NanoDrop spectrophotometer.

### Nucleic acid labeling and IJISS array hybridizations

Immunoprecipitated and input control DNA were labeled with Cy3-dCTP and Cy5-dCTP, respectively, using the BioPrime kit (Invitrogen, Paisley, UK). DNA (0.1-1 μg) was denatured at 94°C for 3 minutes in 40 μl including 20 μl 2.5× random primer mix and kept on ice. Nucleotide mix (5 μl; 2 mM dATP, 2 mM dGTP, 2 mM dTTP, 0.5 mM dCTP), 3.75 μl Cy3/Cy5-dCTP (Perkin Elmer, Beaconsfield, Bucks, UK) and 1.5 μl of Klenow fragment were added and the reaction was incubated at 37°C overnight. The labeled DNA was purified using the MinElute PCR purification kit (Qiagen, Crawley, West Sussex, UK) and the incorporated Cy3/Cy5-dCTP was quantified with the NanoDrop ND-1000 spectrophotometer. For gene expression analysis, cDNA synthesis and labeling were conducted as described previously [62].

For hybridization on Sco-Chip^2^-v1 arrays, 40 pmol of Cy3-labeled immunoprecipitated DNA was co-hybridized with the same amount of Cy5-labeled total input chromatin DNA in 500 μl Agilent hybridization buffer (1 M NaCl, 50 mM MES, pH 7, 20% formamide, 1% Triton X-100 buffer), in an Agilent Technologies hybridization chamber, rotated at 55°C for 60 h in an Agilent Technologies hybridization oven. For hybridization on Sco-Chip^2^-v2 arrays, 10-40 pmol of Cy3-labeled immunoprecipitated DNA were co-hybridized with the same amount of Cy5-labeled control mock IP DNA in 120 μl Agilent hybridization buffer as above. To control for Cy-dye bias, the hybridization was repeated with the same IP DNA samples labeled in the opposite dye orientation. Two biological replicates were hybridized on both array formats.

The arrays were washed once in 50 ml of 6 × SSPE, 0.005% N-lauryl sarcosine and once in 0.06 × SSPE, 0.18% polyethylene glycol 200, both for 5 minutes at room temperature. The arrays were briefly immersed in Agilent Technologies stabilization and drying solution prior to processing in an Agilent Technologies scanner. The probe signals were quantified using Agilent's Feature Extraction software (version 9.1.3.1).

Two different types of dual hybridizations were conducted on the arrays. With the Sco-Chip^2^-v1 arrays, HspR-IP chromatin was co-hybridized with Cy5-labeled total input chromatin as reference and the mock 'no-antibody' IP chromatin was also co-hybridized with total input chromatin on a separate array; the enrichment ratios for each probe were calculated as the signal from the former divided by that from the latter array. With Sco-Chip^2^-v2, the HspR-IP chromatin was co-hybridized directly with the mock 'no antibody' IP chromatin - the sample processed in the same way as the HspR-IP, but without the specific antibody. To compensate for any dye bias in the latter experiments, replicate hybridizations were conducted with both Cy3/Cy5 dye orientations on different arrays. It should be noted that the experimental design in terms of hybridizations are different for Sco-Chip^2^-v1 (IP or mock IP versus total chromatin) and Sco-Chip^2^-v2 (IP versus mock IP), being comparable, respectively, to 'traditional' microarray (expression) experimental designs of indirect and direct hybridizations. However, both designs are valid since ultimately the same output ratio of interest (IP/mock IP signal) is calculated. The direct hybridization approach, introduced for Sco-Chip^2^-v2, is preferred because there is likely to be a reduction in variance (as fewer arrays are used to derive the same ratios).

### RNA isolation analysis, cDNA synthesis and labeling

The RNA preparations used during this study were the same as those reported in [27]. The RNA was isolated from 36 h *S. coelicolor *MT1110 cultures grown on SMMS agar, by the method reported previously [57]; RNA from YEME plus 10% sucrose was isolated from 40 h batch cultures by the RNeasy method described in [27]. RNA quality and integrity was re-assessed using the Agilent Bioanalyzer 2100 system. The Cy3/Cy5-dCTP labeled cDNA was synthesized from 10 μg RNA samples following the methods described in [27].

### Microarray data pre-processing

All (ChIP-on-chip and expression) hybridized arrays were scanned using an Agilent Technologies microarray scanner and resultant intensities calculated using Agilent Technologies Image Analysis and Feature Extraction software (version 9.1.3.1) with local background correction. Log_2 _signal/reference ratios were calculated for all arrays, the reference channel either representing total input chromatin (Sco-Chip^2^-v1 array), mock 'no-antibody' IP (Sco-Chip^2^-v2 array) or cDNA (Sco-Chip^2^-v2 array).

ChIP-on-chip array data were not normalized as the typical microarray normalization assumptions do not hold [63]. All expression arrays were normalized by the Loess method using the LIMMA package [64,65] in R (version 2.5.0 [66,67]). For the heat-shock experiment, across array normalization was applied such that the median absolute deviations (MADs) were similar (scale function in LIMMA); no further normalization was applied to the data from the *hspR *mutant/wild-type comparison.

Within the output of the Feature Extraction software (Agilent Technologies) there are four binary (1 for bad, 0 for good) variables (gIsFeatNonUnifOL/rIsFeatNonUnifOL, gIsBGNonUnifOL/rIsBGNonUnifOL, gIsFeatPopnOL/rIsFeatPopnOL and gIsBGPopnOL/rIsBGPopnOL) that describe outliers in each channel on an array. Spots on the Sco-Chip^2^-v1 array were flagged as poor quality if at least one of the four Feature Extraction variables for the total input chromatin reference channel had the value 1 (bad). Spots on each of the Sco-Chip^2^-v2 arrays were flagged if at least one of the two channels was classed as an outlier by Feature Extraction.

All data were filtered such that only those probes were retained for analysis that had good quality data (not flagged) in each replicate array (to control dye bias) within each independent experiment: 20,586 probes for Sco-Chip^2^-v1 array; 43,056 probes for Sco-Chip^2 ^(ChIP-on-chip); and 43,263 probes for the Sco-Chip^2^-v2 gene expression analysis.

### Microarray expression data analysis

The filtered data sets for the gene expression experiments were analyzed using rank products analysis [37] via the RankProd package in R (version 2.5.0) [68]. This method has been shown to be robust in the identification of true differentially expressed genes in data sets where there are few replicates and/or large variance [69,70]. Differentially expressed genes were identified as having a pfp value [37] less than or equal to 0.15, equal to a false discovery rate of approximately 15%, a threshold value lower than that applied in the literature with this technique [37]. The microarray-derived expression data have been deposited in ArrayExpress (accession numbers [E-MAXD-44], [E-MAXD-46] and [E-MAXD-49]).

### ChIP-on-chip data analysis

#### Sco-Chip^2^-v1 array

Genes were identified as having upstream regions enriched/bound by HspR by the following steps. Step 1, probes that had a significant (corrected *p*-value < 0.05, non-parametric *t*-test [71] (using Bioconductor package [65])) difference between log_2 _'antibody IP'/total chromatin and log_2 _'no-antibody IP'/total chromatin were identified. Step 2, the average (across biological replicates) log_2 _antibody IP/total chromatin - log_2 _no antibody IP/total chromatin distribution of significant probes was plotted. Step 3, the right tail, the region of the distribution that departs from the typical Gaussian curve of the distribution, was identified and used as the threshold (Additional data file 1). Step 4, enriched probes were identified as having a ratio (average log_2 _antibody IP/total chromatin - log_2 _no antibody IP/total chromatin) > threshold (Figure 2a). Step 5, genes that had at least two probes enriched in a promoter proximal region were scored as likely targets of HspR (Additional data file 3).

#### Sco-Chip^2^-v2 array

Genes/regions considered to be directly controlled by HspR were first identified using steps 1-4 (see Additional data file 2 and Figure 3) as described above with the following exceptions: significant probes were determined by the difference between log_2 _'HspR antibody IP'/'no-antibody IP' and log_2 _'HspR pre-immune serum antibody IP'/'no-antibody IP' (step 1); average (across biological replicates) log_2 _'HspR antibody IP'/'no-antibody IP' distribution of significant probes was plotted (step 2) to determine the threshold (>1.5 on log_2 _scale) of selection (step 3). Then the novel approach, for ChIP-on-chip analysis, of clustering enriched probes was undertaken as follows such that identified probe clusters corresponded to the most likely targets of HspR. Steps 1-4 were as above. Step 5, enriched probes were represented by their corresponding position in the genome. A distance matrix was constructed based on the Euclidean distance between each pair (within the enriched probes set) of probe positions, when represented on a log scale. Step 6, the distance matrix was normalized such that each distance was between 0 and 1 and converted into a similarity matrix by subtracting each normalized distance from 1. Thus, probes with a similarity score of 0 are distant from each other in the genome and probes with a similarity score close to 1 are near each other in the genome. Step 7, the similarity matrix was given as input to the clustering algorithm CAST (Clustering Affinity Search Technique) [72], which intrinsically calculates the optimal partitioning of a data set (via addition and removal of members to a cluster such that the affinity of a cluster remains 'tight') given a certain threshold. The threshold of 0.5 was used to cluster probes; this threshold is equivalent to grouping enriched probes that occur within 2.9 kb of each other in the genome. Step 8, clusters of size greater than 1 were annotated with gene names (SCO numbers) and designated as genes/regions bound by HspR.

The ChIP-on-chip data have been deposited in ArrayExpress (accession number [E-MAXD-48]).

### Nucleotide sequence motif analysis

#### Identification of putative HspR target sites with HspR consensus sequence

For each gene identified experimentally as having a putative HspR binding site, the respective upstream sequence, 300 nucleotides upstream of the translational start codon and 200 nucleotides downstream of the translational start codon, in the correct transcriptional orientation, was extracted from the annotated genome file of *S*. *coelicolor*. The entire set of upstream sequences was then aligned independently to the previously published HspR consensus sequence (5'-TTGAGYN(7)ACTCAA) [6] using ClustalW [73].

#### Derivation of a new HspR consensus sequence

The set of upstream sequences representing putative HspR binding sites was submitted to the MEME (Multiple Em for Motif Elicitation) server [74,75] using default settings except for: minimum width of the motif to search for was set to 5 nucleotides and maximum width to 30 nucleotides (to reflect the common transcription factor recognition site length in prokaryotes); and maximum number of sites to find was set to 10, to restrict the amount of data obtained.

#### Quantitative real time PCR analysis of selected differentially expressed genes

Specific primers and probes for *SCO4410*, *SCO5639*, *SCO3202 *and *SCO4157 *were designed using Primer 3 software and used for qPCR as described previously [7]. The sequences for the forward and reverse primers and dual labeled probes were: *SCO4410 *forward primer 5'-GTGTCGGGCGAACTGG, reverse primer 5'-CCGGGACGCGATGA, and dual labeled probe 5'-TCTGCGATTCCAGCGGGGTC; *SCO5639 *forward primer 5'-ACCATGAAGACGAGAGAGAGG, reverse primer 5'-GTGCACGAACACGTCT, and dual labeled probe 5'-ATGCCGGGCGACGTGCTAAA; *SCO3202 *forward primer 5'-CTGATCCAGGAGGGCAAC, reverse primer 5'-GCGTACGTGGAGAACTTGAA, and dual labeled probe 5'-TCCGCGCGGTCGAGAAGTTC; *SCO4157 *forward primer 5'-GACGTACAAGGCGATCCAG, reverse primer 5'-ATGATGTTGCCGTTCATGTC, and dual labeled probe 5'-CCCTCAACCCGGGCAACTCC.

### Electrophoretic mobility shift assays

Gel-shift assays were conducted using the Light Shift Chemiluminescence EMSA kit (Pierce), following the manufacturer's instructions. The 3'-biotinylated oligonucleotides comprising the HspR binding site, IR3 [26] in the promoter region of the *dnaK *operon, and similar motifs in the promoter regions of *SCO5639 *and *SCO4410 *were annealed to their complementary strands and 200 fmol were used in the binding reaction together with either purified DnaK-refolded HspR (as described in [61]) or a JM109 *E. coli *cell extract over-expressing *hspR *[26]. In competition experiments a 200-fold excess of the same, but non-biotinylated, DNA was included in the electrophoretic mobility shift assay reaction. The *dnaK *biotinylated probe used in gel shift assays was 5'-TGCACACTTGAGCCTGTTCCACTCAAGTCAGCTGGAG; the *SCO5639 *biotinylated probe was 5'-TCGGATTGGAATTACTAAGATTCAGGATGCAGCACGCATCGT and the *SCO4410 *biotinylated probe was 5'-CGTTTCGGGTGAATCCCGAAAATTCCAGACGTTCCGACGAGG.

## Abbreviations

ChIP-on-chip: chromatin immunoprecipitation-on-chip; Cy3: cyanine-3 dye; Cy5: cyanine-5 dye; IP: immunoprecipitation; IJISS: ink-jet *in situ*-synthesized; MEME: Maximum Em for Motif Elicitation; pfp: probability of false prediction; qPCR: quantitative real time PCR.

## Authors' contributions

GB performed the microbiological and molecular biology work, including most of the ChIP method development and microarray-based experiments. EL participated in the array design, developed the new ChIP-chip data clustering method, conducted the microarray data/sequence analysis and other statistical studies. VM and DH contributed to microarray validation and method development. NA contributed to method development. JH, VB and MH conducted the probe design, final probe selection and co-ordinated microarray fabrication. MH co-ordinated the array design activities of Oxford Gene Technology Ltd. CPS conceived of the study and participated in its design and coordination. GB, EL and CPS wrote the manuscript.

## Additional data files

The following additional data are available with the online version of this paper. Additional data file 1 illustrates the distribution of significant probes from Sco-Chip^2^-v1 arrays. Additional data file 2 illustrates the distribution of significant probes from Sco-Chip^2^-v2 arrays. Additional data file 3 illustrates probe signals across significantly scoring HspR target regions using the Sco-Chip^2^-v1 array. Additional data file 4 tabulates genes significantly up-regulated following heat-shock at 42°C. Additional data file 5 lists the genes up-regulated in an *hspR *disruption mutant. Additional data file 6 illustrates the partial matches to the HspR-binding consensus sequence within the five tRNA^Gln/Glu ^cluster. Additional data file 7 details the motif identified from MEME analysis of the HspR targets identified with the Sco-Chip^2^-v2 array. Additional data file 8 tabulates amino acid composition of heat-shock-induced gene products relative to all annotated proteins. Additional data file 9 details qPCR expression data for selected heat induced genes. Additional data file 10 shows the qPCR expression data for *SCO4410 *and *SCO5639*. Additional data file 11 shows the comparison of heat shock expression data in SMMS and YEME cultures. Additional file 12 lists probes found to be significantly enriched for HspR on Sco-Chip^2^-v1 arrays. Additional file 13 lists probes found to be significantly enriched for HspR on Sco-Chip^2^-v2 arrays. Additional data file 14 shows an enlarged image of Figure 3d. Additional data file 15 shows an enlarged image of Figure 3e.

## Supplementary Material

Additional data file 1 Average (across biological replicates) plotted as [log_2 _Antibody bound/Total chromatin - log_2 _'No Antibody' bound/Total chromatin]. The red line indicates the threshold applied to identify enriched probes. Click here for file

Additional data file 2 Average (across biological replicates) plotted as [log_2 _Antibody bound/'No Antibody' bound]. The red line indicates the threshold applied to identify enriched probes. Click here for file

Additional data file 3 The anti-HspR-enriched probes are plotted on a linear scale. Open circles indicate the start co-ordinate (relative to genome sequence) of each probe that passed quality control filtering. The genetic organization of each region is indicated below the plot; each arrow represents a coding sequence or stable RNA gene as defined in [EMBL:AL645882.2]. Click here for file

Additional data file 4 Tabulation of genes significantly up-regulated following heat-shock at 42°C. Click here for file

Additional data file 5 Tabulation of genes significantly up-regulated in an *hspR *disruption mutant relative to wild-type. Click here for file

Additional data file 6 Partial matches to the HspR-binding consensus sequence within the five tRNA^Gln/Glu ^cluster. Click here for file

Additional data file 7 Motif identified from MEME analysis of the HspR targets identified with the Sco-Chip^2^-v2 array. Click here for file

Additional data file 8 Over-representation of amino acids in heat-shock induced gene products (listed in Additional data file 4) relative to all annotated proteins from the *S. coelicolor *genome [EMBL: AL645882.2]. Click here for file

Additional data file 9 *SCO3202 *(*hrdD*) and *SCO4157 *had respective rank products pfp values of 0.12 and 0.13. *SCO4410 *and *SCO5639 *were identified as new putative targets for HspR. Average values are plotted from the same two independent biological replicates of each condition used in the array-based expression analysis. Click here for file

Additional data file 10 Average values are plotted from the same two independent biological replicates of each condition used in the array-based expression analysis. Click here for file

Additional data file 11 Comparison of expression of heat-shock genes from SMMS surface-grown cultures (Additional data file 4) in YEME+10.3% sucrose liquid cultures (as used in ChIP-on-chip experiments). Click here for file

Additional data file 12 Probes found to be significantly enriched for HspR on Sco-Chip^2^-v1 arrays. Click here for file

Additional data file 13 Probes found to be significantly enriched for HspR on Sco-Chip^2^-v2 arrays. Click here for file

Additional data file 14 An enlarged image of Figure 3d (*rrnD *data), with a dotted line along log ratio of 0. Click here for file

Additional data file 15 An enlarged image of Figure 3e (tRNA cluster data), with a dotted line along log ratio of 0. Click here for file
