# Supplementary figures and images for: Development and application of versatile high density microarrays for genome-wide analysis of Streptomyces coelicolor: characterization of the HspR regulon
Source: Genome Biol. 2009 Jan 16;10(1):R5. doi: 10.1186/gb-2009-10-1-r5 (PMC2687793; doi:10.1186/gb-2009-10-1-r5)

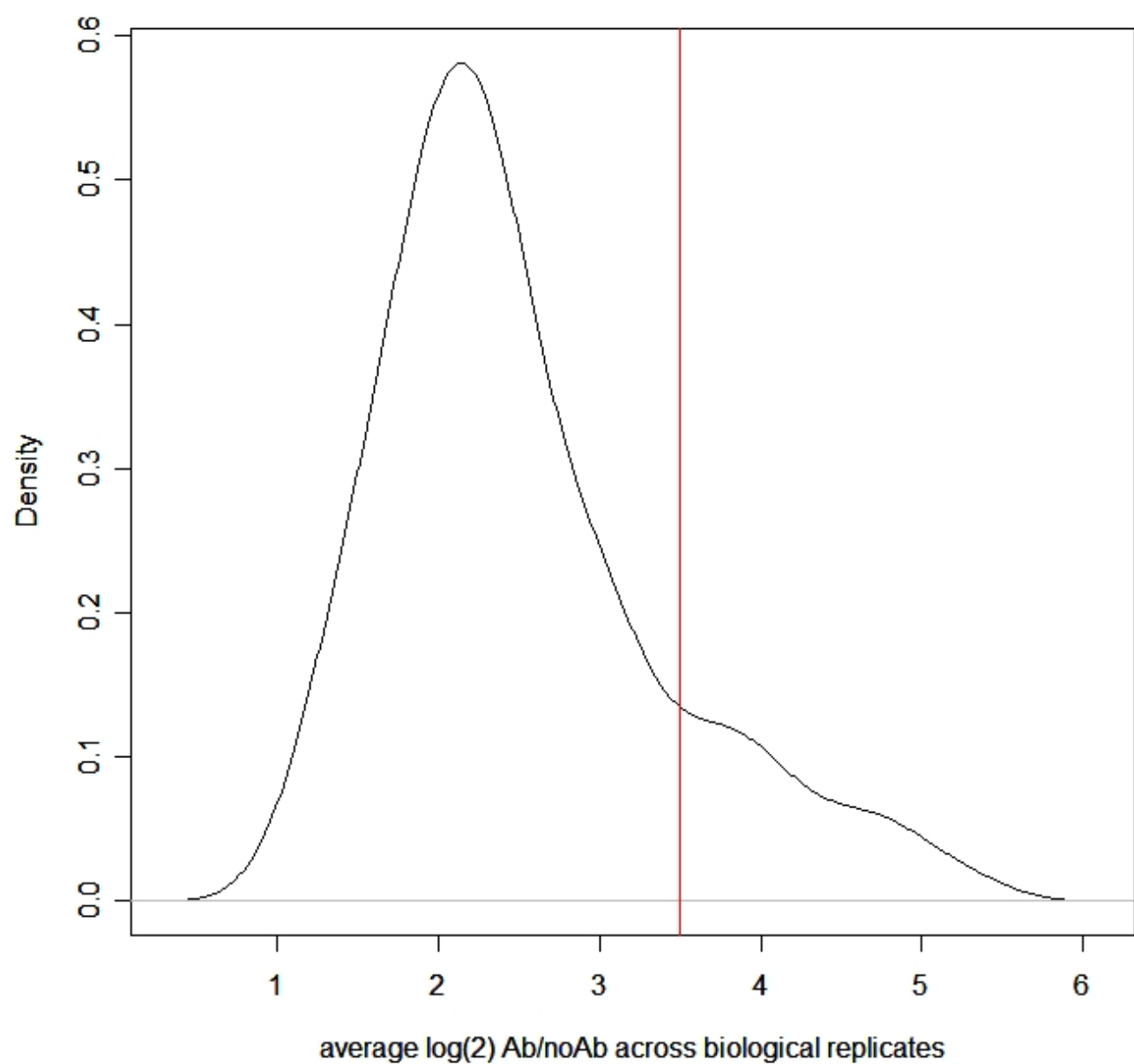

Supplement: Additional data file 1 — Average (across biological replicates) plotted as [log2 Antibody bound/Total chromatin - log2 'No Antibody' bound/Total chromatin]. The red line indicates the threshold applied to identify enriched probes. [file gb-2009-10-1-r5-S1.pdf]

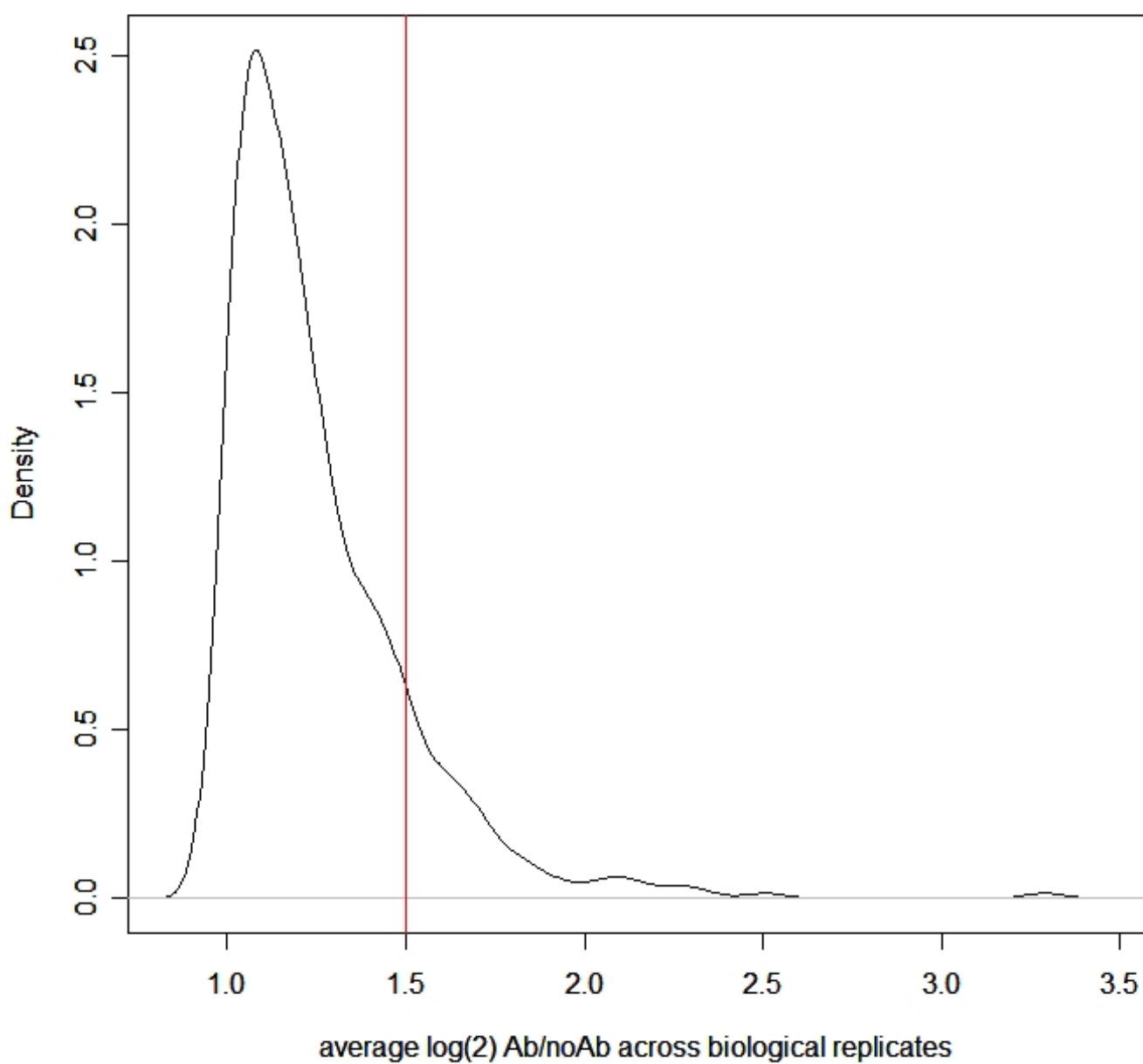

Supplement: Additional data file 2 — Average (across biological replicates) plotted as [log2 Antibody bound/'No Antibody' bound]. The red line indicates the threshold applied to identify enriched probes. [file gb-2009-10-1-r5-S2.pdf]

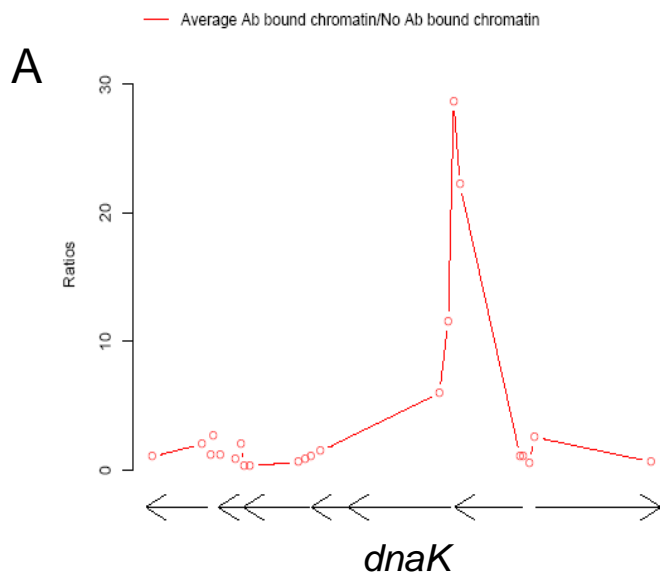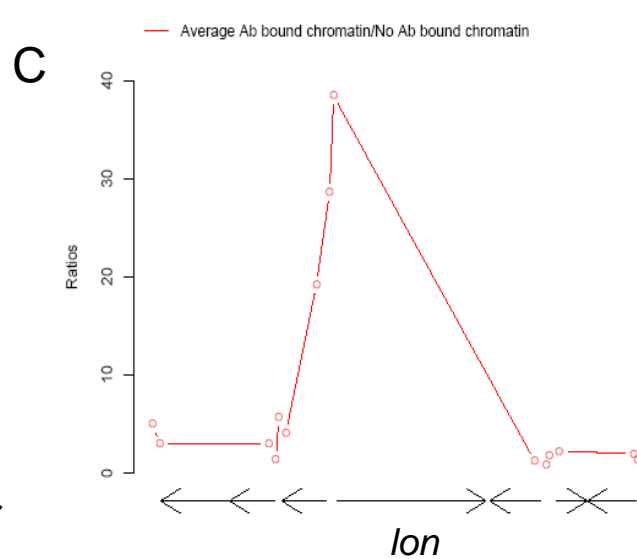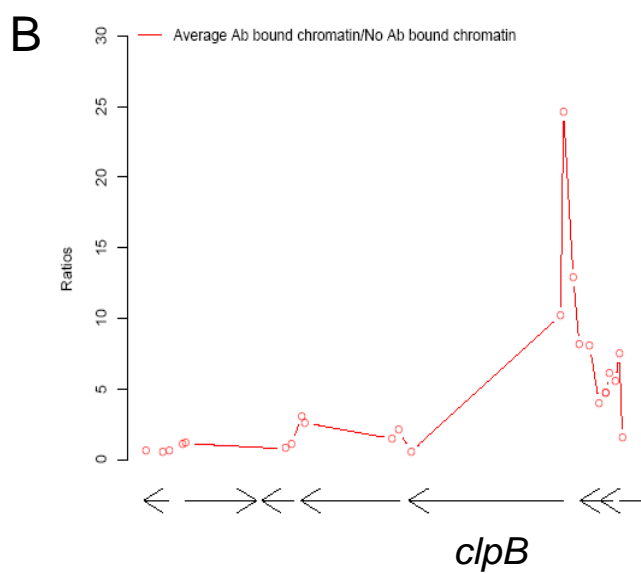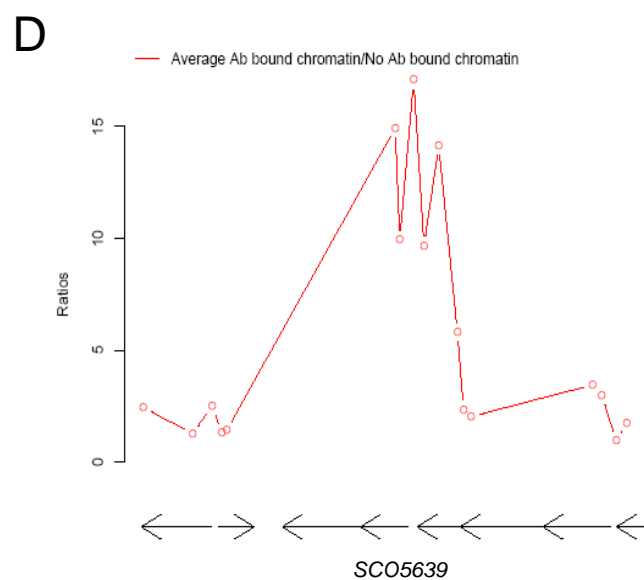

Supplement: Additional data file 3 — The anti-HspR-enriched probes are plotted on a linear scale. Open circles indicate the start co-ordinate (relative to genome sequence) of each probe that passed quality control filtering. The genetic organization of each region is indicated below the plot; each arrow represents a coding sequence or stable RNA gene as defined in [EMBL:AL645882.2]. [file gb-2009-10-1-r5-S3.pdf]

Positions of partial HspR motif sequences in the five tRNA Gln/Glu cluster

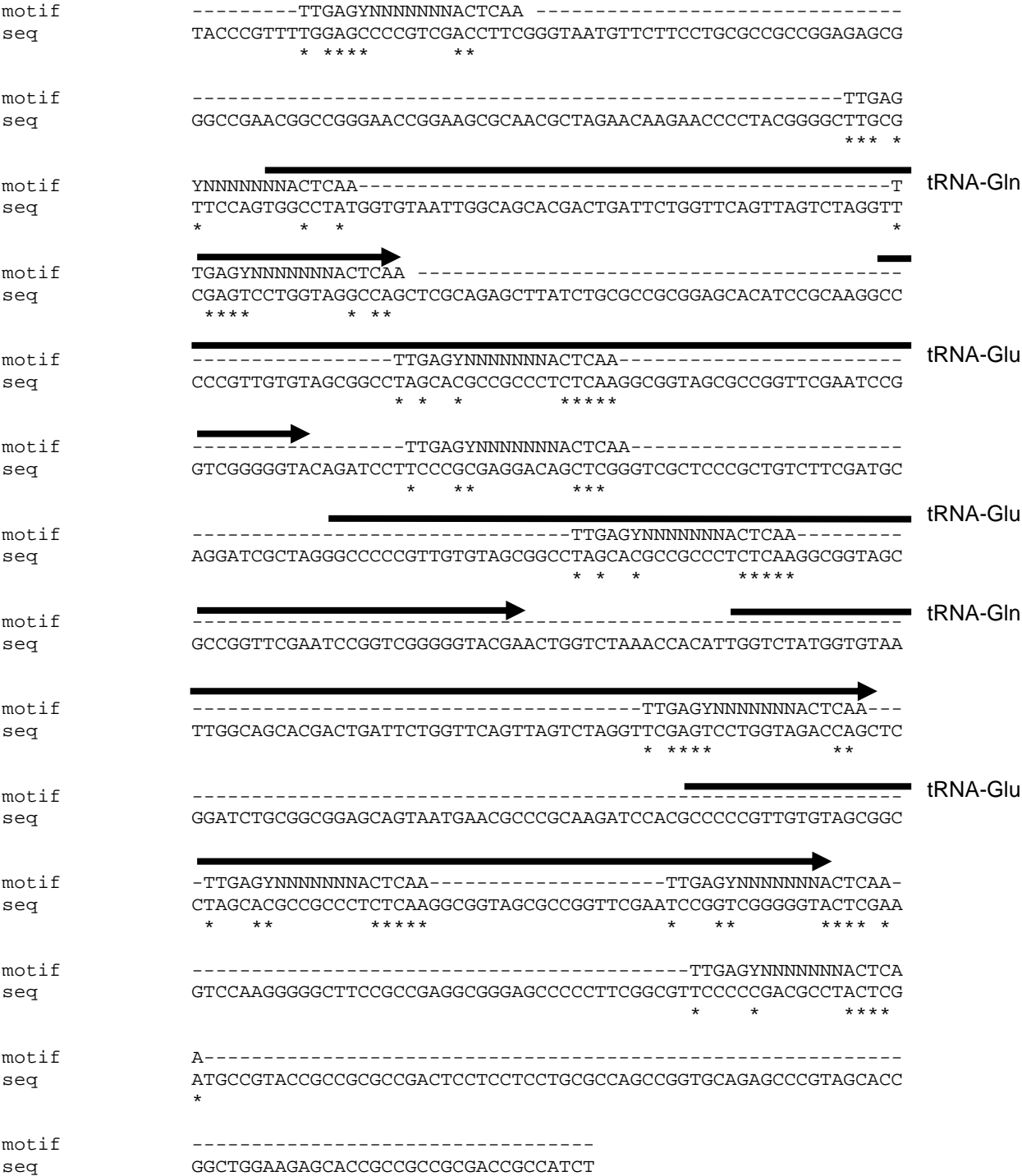

Supplement: Additional data file 6 — Partial matches to the HspR-binding consensus sequence within the five tRNAGln/Glu cluster. [file gb-2009-10-1-r5-S6.pdf]

SCO4157

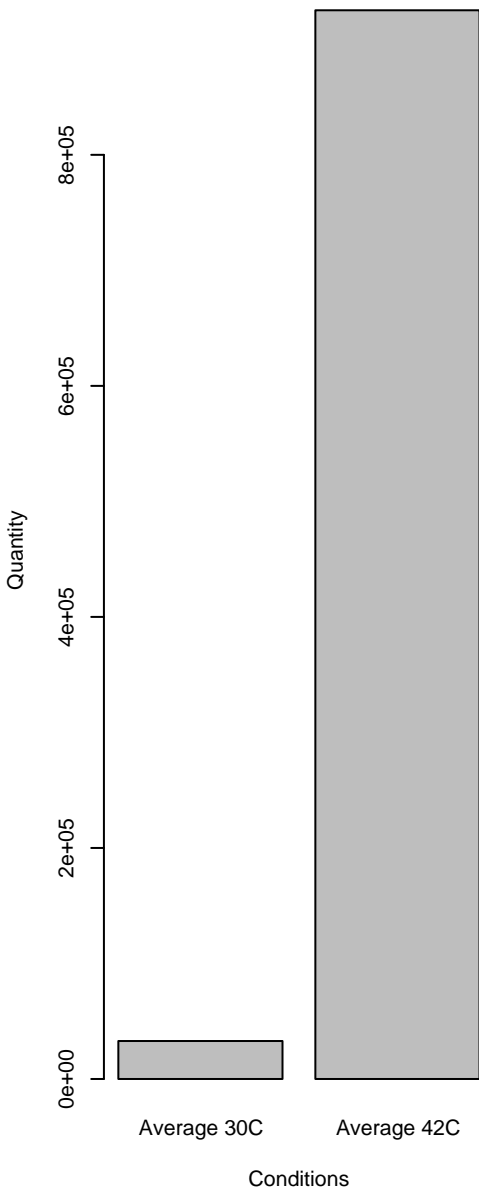

SCO3202

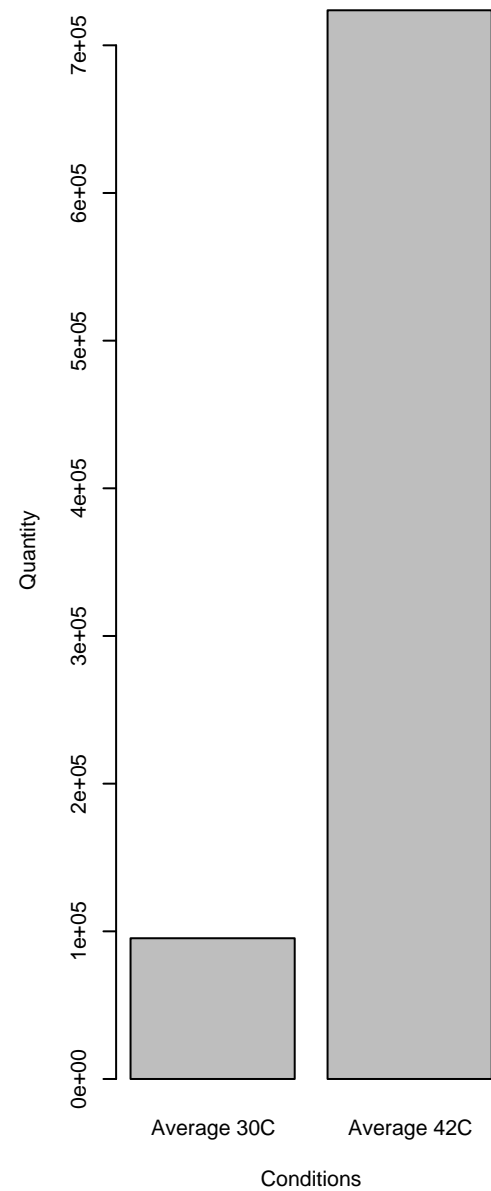

SCO3671 (dnaK)

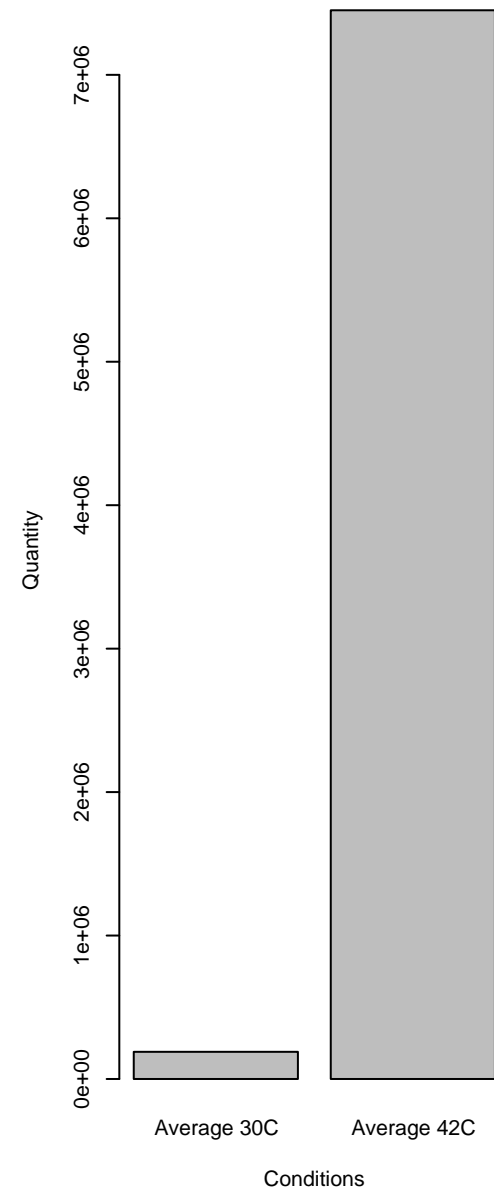

SCO4410

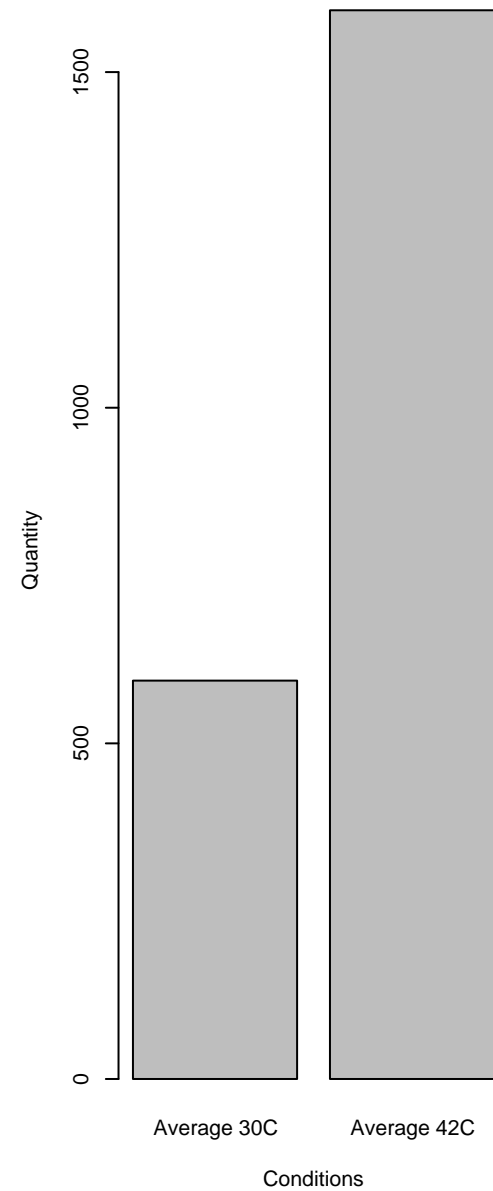

SCO5639

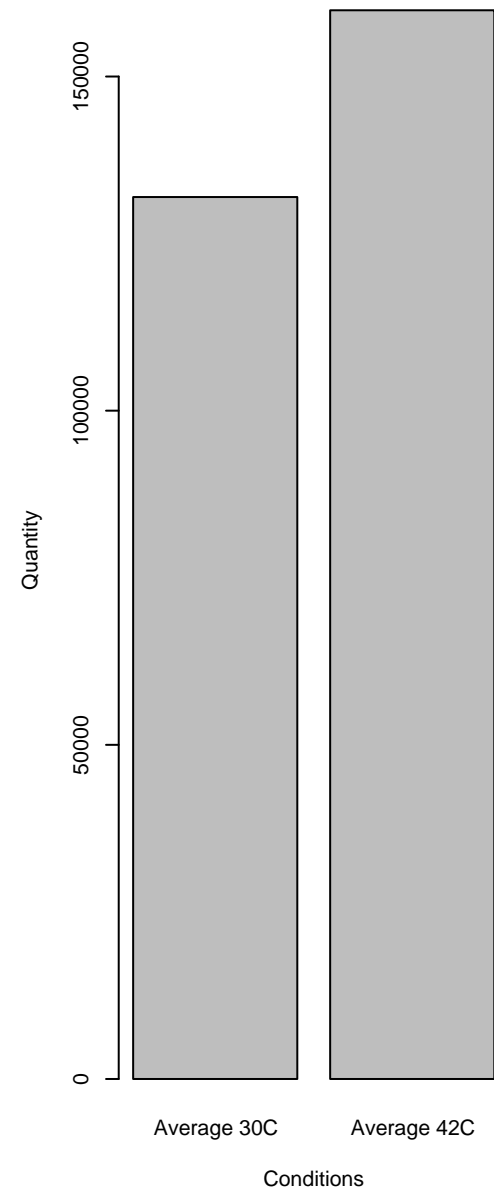

Supplement: Additional data file 9 — SCO3202 (hrdD) and SCO4157 had respective rank products pfp values of 0.12 and 0.13. SCO4410 and SCO5639 were identified as new putative targets for HspR. Average values are plotted from the same two independent biological replicates of each condition used in the array-based expression analysis. [file gb-2009-10-1-r5-S9.pdf]

SCO4410

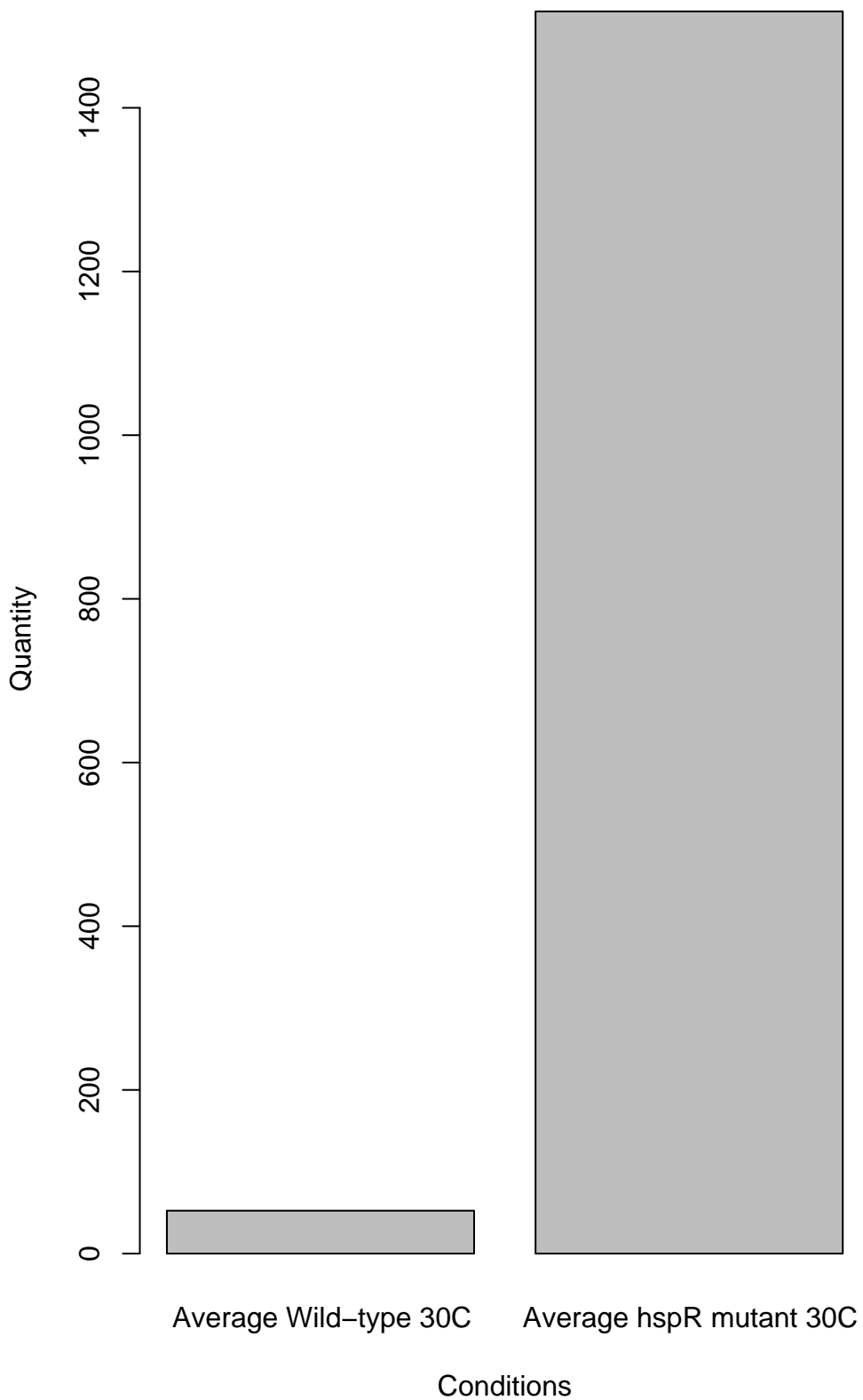

SCO5639

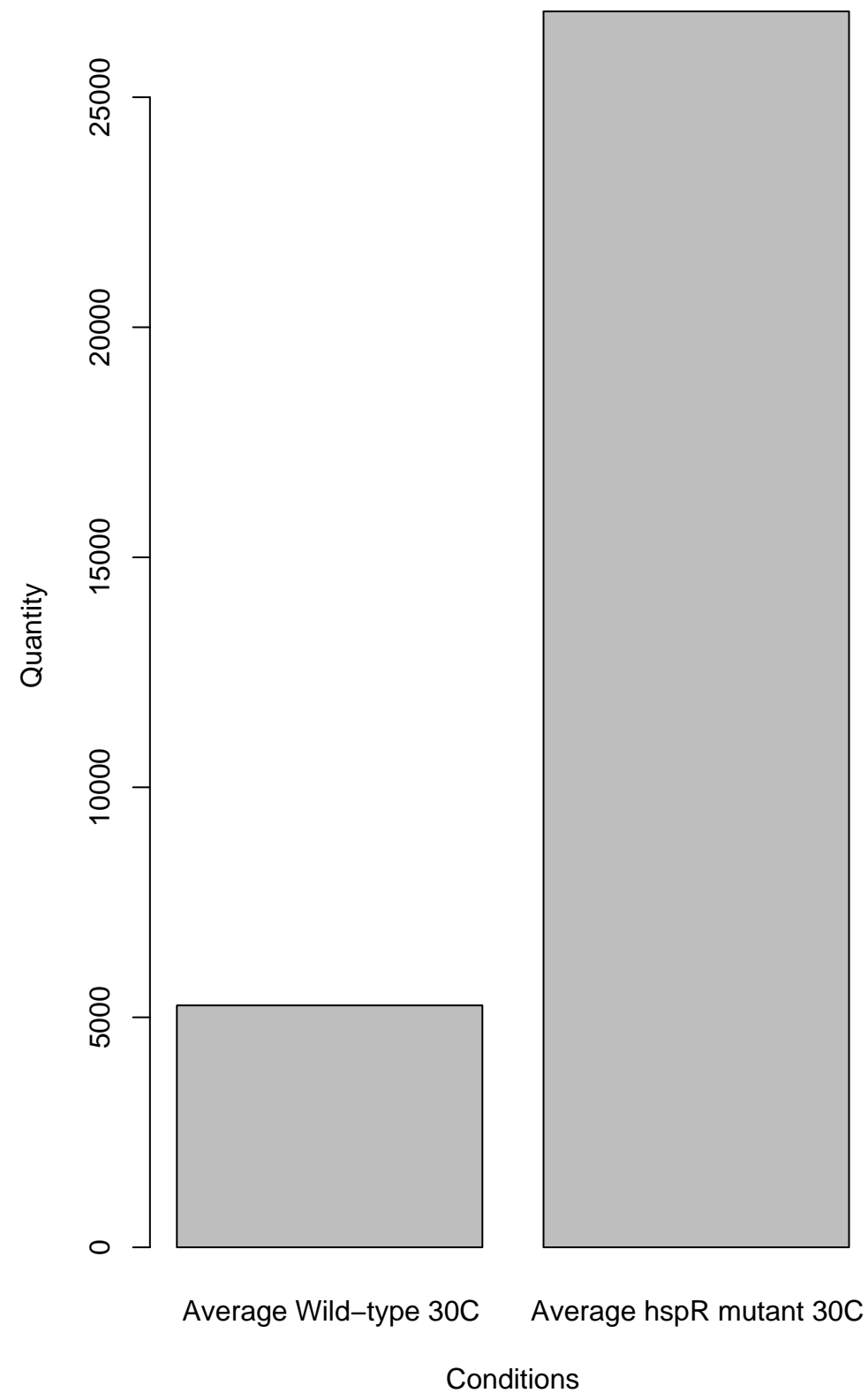

Supplement: Additional data file 10 — Average values are plotted from the same two independent biological replicates of each condition used in the array-based expression analysis. [file gb-2009-10-1-r5-S10.pdf]

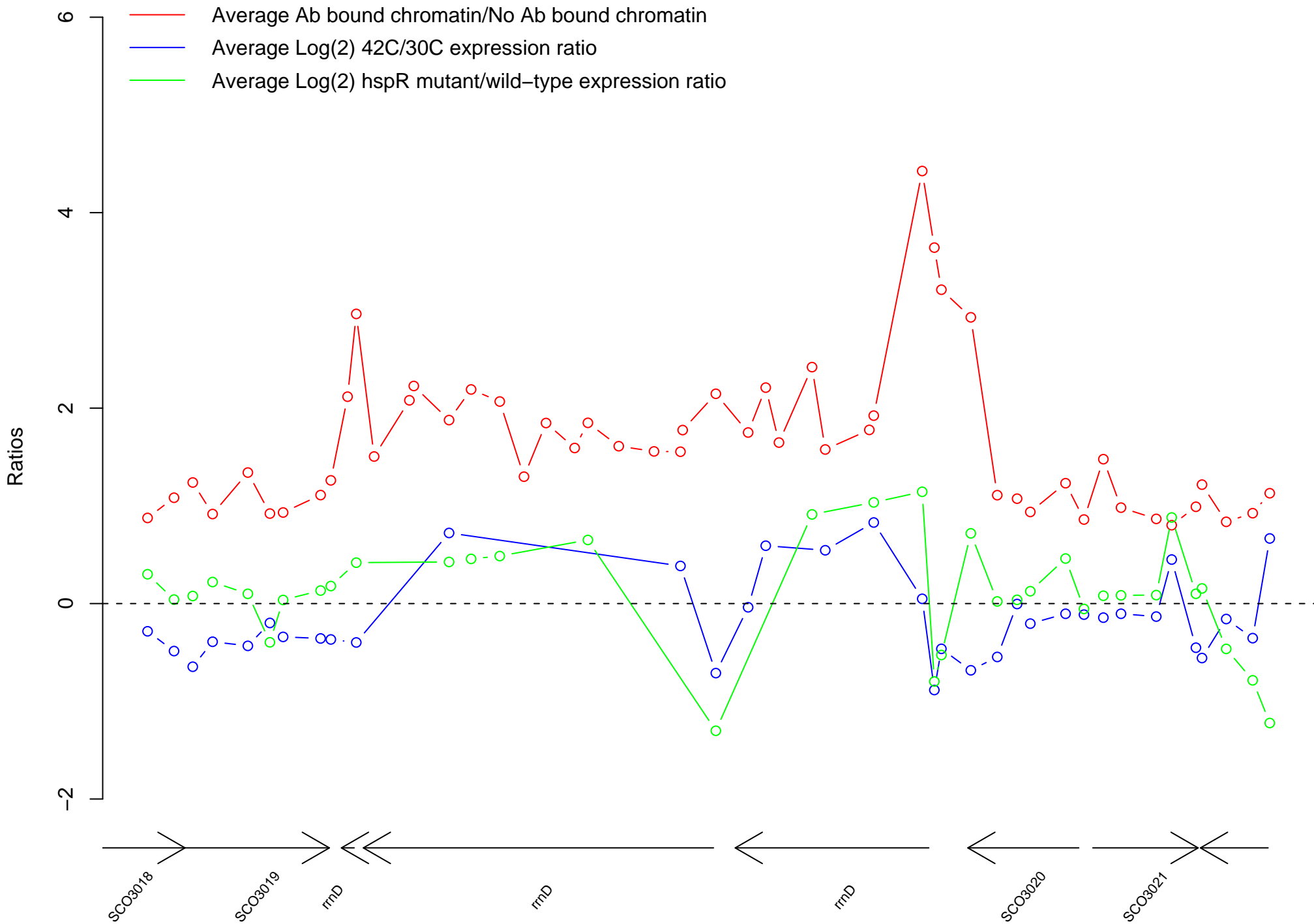

Supplement: Additional data file 14 — An enlarged image of Figure 3d (rrnD data), with a dotted line along log ratio of 0. [file gb-2009-10-1-r5-S14.pdf]

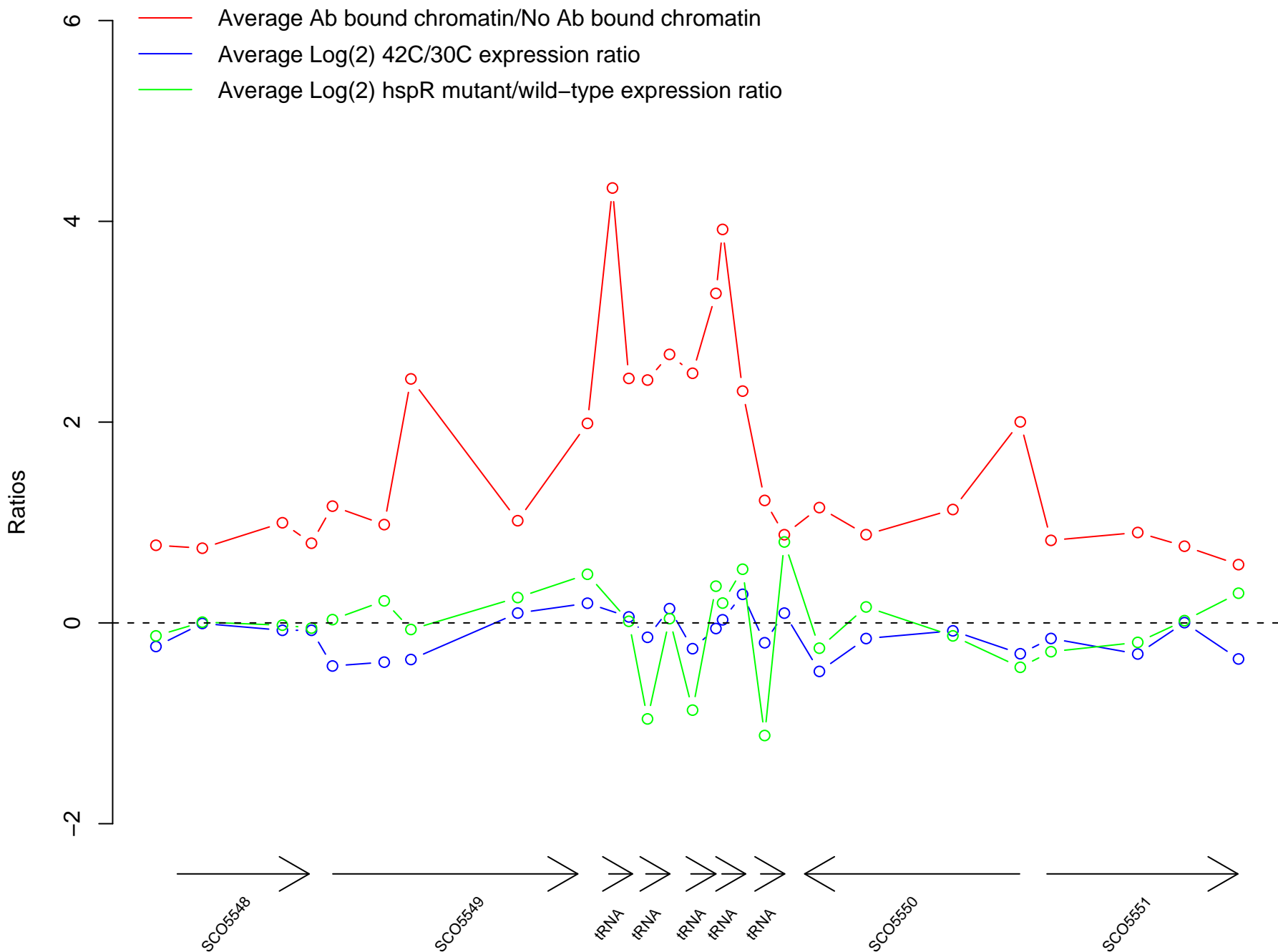

Supplement: Additional data file 15 — An enlarged image of Figure 3e (tRNA cluster data), with a dotted line along log ratio of 0. [file gb-2009-10-1-r5-S15.pdf]
