# Supplementary material for: Development and application of versatile high density microarrays for genome-wide analysis of Streptomyces coelicolor: characterization of the HspR regulon
Source: Genome Biol. 2009 Jan 16;10(1):R5. doi: 10.1186/gb-2009-10-1-r5 (PMC2687793; doi:10.1186/gb-2009-10-1-r5)
Supplement: Additional data file 4 — Tabulation of genes significantly up-regulated following heat-shock at 42°C. [file gb-2009-10-1-r5-S4.pdf]

Additional File 4 - The heat-shock stimulon of *Streptomyces coelicolor*
Genes are ranked by physical order within genome. Significance cut-off of pfp < 0.15

Gene	Average log(2) 42°C /30 ^{o}C	pfp value	Annotation
SCO0117	3.72	0.02	putative oxidoreductase
SCO0162	3.19	0.07	conserved hypothetical protein
SCO0167	3.47	0.04	conserved hypothetical protein
SCO0168	3.81	0.02	possible regulator protein
SCO0169	3.26	0.06	conserved hypothetical protein
SCO0173	2.49	0.14	hypothetical protein
SCO0174	4.27	0.01	putative DNA-binding protein
SCO0177	3.24	0.06	putative membrane protein
SCO0179	2.34	0.13	putative zinc-containing dehydrogenase
SCO0181	3.38	0.04	hypothetical protein
SCO0199	3.51	0.03	putative alcohol dehydrogenase
SCO0200	3.60	0.03	conserved hypothetical protein
SCO0201	2.96	0.09	putative integral membrane protein
SCO0208	2.38	0.15	pyruvate phosphate dikinase
SCO0210	2.51	0.13	hypothetical protein
SCO0212	2.93	0.09	hypothetical protein
SCO0213	2.62	0.13	putative nitrate/nitrite transporter protein
SCO0214	2.72	0.11	conserved hypothetical protein
SCO0216	3.03	0.09	narG2,nitrate reductase alpha chain
SCO0217	2.83	0.12	narH2,nitrate reductase beta chain
SCO0219	3.51	0.03	narI2,putative nitrate reductase delta chain
SCO0463	2.66	0.09	conserved hypothetical protein
SCO0596	3.43	0.03	putative DNA-binding protein
SCO0600	2.95	0.11	sig8,RNA polymerase sigma factor sig8
SCO0678	4.25	0.01	hypothetical protein
SCO0679	3.21	0.06	hypothetical protein
SCO0761	2.83	0.09	hypothetical protein
SCO0776	2.88	0.09	putative integral membrane protein
SCO0779	2.44	0.14	conserved hypothetical protein
SCO0784	2.90	0.09	putative secreted protein
SCO1002	4.57	0.00	hypothetical protein
SCO1131	3.27	0.04	conserved hypothetical protein
SCO1132	4.46	0.01	putative oxidoreductase
SCO1133	4.29	0.01	putative oxidoreductase, molybdopterin binding subunit
SCO1134	4.64	0.01	putative oxidoreductase, iron-sulphur binding subunit
SCO1225	2.67	0.13	putative osmoprotectant transporter
SCO1373	2.25	0.14	conserved hypothetical protein
SCO2047	2.47	0.12	putative DNA-binding protein
SCO2055	2.86	0.07	hypothetical protein
SCO2256	3.17	0.05	panB,3-methyl-2-oxobutanoate hydroxymethyltransferase
SCO2315	3.63	0.03	putative membrane protein
SCO2341	3.36	0.04	hypothetical protein
SCO2342	2.50	0.13	putative secreted protein
SCO2645	4.12	0.02	putative integral membrane protein
SCO2646	4.45	0.01	putative integral membrane protein
SCO2811	2.33	0.12	putative regulator
SCO2828	2.59	0.12	probable amino acid ABC transporter protein, solute-binding component.
SCO2829	2.63	0.11	probable amino acid ABC transporter protein, integral membrane component.

SCO2830	2.40	0.13	probable amino acid ABC transporter protein, integral membrane component.
SCO2928	2.36	0.11	putative asnC-family transcriptional regulator
SCO3105	2.53	0.12	hypothetical protein
SCO3106	2.41	0.14	putative lipoprotein
SCO3158	2.74	0.09	hypothetical protein
SCO3187	2.88	0.08	conserved hypothetical protein
SCO3202	2.54	0.12	hrdD,RNA polymerase principal sigma factor
SCO3356	2.31	0.13	sigE, ECF sigma factor
SCO3357	2.25	0.13	cseA, lipoprotein
SCO3537	2.68	0.11	putative DNA-binding protein
SCO3538	2.48	0.13	hypothetical protein
SCO3660	2.67	0.12	hypothetical protein
SCO3661	4.11	0.02	clpB, ATP-dependent protease ATP-binding subunit
SCO3668	3.53	0.03	hspR,putative heat shock protein
SCO3669	3.70	0.03	dnaJ,molecular chaperone
SCO3670	4.63	0.01	grpE,heat shock protein
SCO3671	4.86	0.01	dnaK,heat shock protein 70
SCO3802	2.91	0.09	putative membrane protein
SCO3937	2.41	0.12	putative integrase /recombinase
SCO3945	3.69	0.03	cydA, putative cytochrome oxidase subunit I
SCO3946	2.82	0.08	cydB, putative cytochrome oxidase subunit II
SCO4157	2.20	0.13	putative protease
SCO4198	2.31	0.14	putative DNA-binding protein
SCO4296	3.79	0.03	groEL2,chaperonin 2
SCO4615	3.52	0.03	integrase
SCO4616	4.58	0.01	xis,excisionase
SCO4617	3.27	0.04	putative ATP-binding protein
SCO4618	3.78	0.02	hypothetical protein
SCO4619	3.46	0.03	putative integral membrane protein
SCO4620	3.14	0.04	traB1,putative sporulation-related protein
SCO4621	2.44	0.11	traA1,putative sporulation-related protein
SCO4624	4.65	0.01	hypothetical protein
SCO4625	3.23	0.03	conserved hypothetical protein
SCO4626	3.96	0.01	hypothetical protein
SCO4761	3.75	0.03	groES,10 kD chaperonin cpn10
SCO4762	4.05	0.02	groEL1,60 kD chaperonin cpn60
SCO4903	2.56	0.12	putative membrane protein
SCO5236	2.60	0.11	nagB,putative glucosamine phosphate isomerase
SCO5237	3.39	0.03	putative oxidoreductase
SCO5238	2.21	0.14	putative TetR-family protein
SCO5269	2.17	0.14	hypothetical protein
SCO5285	4.66	0.01	lon,ATP-dependent protease
SCO5848	2.43	0.11	agaZ,tagatose 6-phosphate kinase
SCO5850	2.09	0.14	conserved hypothetical protein
SCO5917	4.08	0.02	conserved hypothetical protein
SCO6174	2.25	0.13	hypothetical protein
SCO6499	4.35	0.01	gvpO,putative gas vesicle synthesis protein
SCO6500	3.58	0.03	gvpA,putative gas vesicle synthesis protein
SCO6502	2.85	0.09	gvpG,putative gas vesicle synthesis protein
SCO6504	3.04	0.09	conserved hypothetical protein
SCO6505	2.70	0.12	gvpJ,putative gas vesicle synthesis protein

SCO6515	3.21	0.05	pfpI ,putative protease
SCO7095	2.87	0.07	putative hydrolase
SCO7100	2.51	0.10	putative oxidoreductase
SCO7101	2.69	0.09	putative dehydrogenase
SCO7189	2.62	0.11	hypothetical protein
SCO7238	3.72	0.03	hypothetical protein
SCO7277	2.55	0.12	putative regulator protein
SCO7278	2.73	0.11	putative RNA polymerase sigma factor
SCO7317	3.60	0.03	hypothetical protein
SCO7325	3.53	0.03	anti-sigma factor antagonist
SCO7442	2.57	0.13	hypothetical protein
SCO7446	2.34	0.13	putative regulator
SCO7646	4.02	0.01	putative membrane protein
SCO7734	2.43	0.13	hypothetical protein
SCO7735	2.36	0.12	hypothetical protein
SCO7747	3.72	0.03	conserved hypothetical protein
SCO7755	2.88	0.09	conserved hypothetical protein
SCO7756	2.61	0.13	hypothetical protein
SCO7809	2.23	0.12	putative TetR-family transcriptional regulator
SCO7821	2.53	0.13	putative integral membrane protein
