# Supplementary material for: Development and application of versatile high density microarrays for genome-wide analysis of Streptomyces coelicolor: characterization of the HspR regulon
Source: Genome Biol. 2009 Jan 16;10(1):R5. doi: 10.1186/gb-2009-10-1-r5 (PMC2687793; doi:10.1186/gb-2009-10-1-r5)
Supplement: Additional data file 5 — Tabulation of genes significantly up-regulated in an hspR disruption mutant relative to wild-type. [file gb-2009-10-1-r5-S5.pdf]

Additional File 5 - Genes up-regulated in an *hspR* disruption mutant of *Streptomyces coelicolor*

Genes are ranked by physical order within genome. Significance cut-off of pfp < 0.15

Gene	Average log(2) WT/hspR mutant	pfp value	Annotation
SCO0037	-2.12	0.04	possible sigma factor
SCO0262	-1.96	0.06	probable LysR-family transcriptional regulator
SCO0627	-1.98	0.06	possible ATP utilising protein
SCO0668	-1.55	0.11	possible flavoprotein
SCO0720	-1.58	0.13	possible integral membrane protein
SCO0984	-1.91	0.07	possible 3-hydroxyacyl-CoA dehydrogenase
SCO1036	-2.67	0.02	probable phosphotriesterase-family protein
SCO1203	-1.83	0.09	possible MutT-like protein
SCO1234	-2.03	0.05	ureC, urease alpha subunit
SCO1454	-1.74	0.11	possible amino oxidase
SCO1457	-1.73	0.11	probable transport protein
SCO1656	-2.71	0.01	possible hydrolase
SCO2029	-2.47	0.02	putative secreted protein
SCO2091	-2.07	0.04	possible membrane protein
SCO2543	-2.10	0.04	possible dihydrodipicolinate synthase
SCO2570	-1.75	0.10	possible secreted protein
SCO2795	-1.72	0.12	probable sugar binding secreted protein
SCO2818	-2.37	0.02	hypothetical protein
SCO3044	-1.64	0.13	conserved hypothetical protein
SCO3097	-2.31	0.03	possible secreted protein
SCO3484	-1.82	0.09	possible secreted sugar-binding protein
SCO3488	-1.69	0.13	probable transposase remnant
SCO3660	-1.62	0.11	hypothetical protein
SCO3661	-2.27	0.03	clpB, probable clp-family ATP-binding subunit
SCO3669	-3.02	0.01	dnaJ, molecular chaperone
SCO3670	-3.84	0.00	grpE, heat shock protein
SCO3671	-4.23	0.00	dnaK, heat shock protein 70 (fragment)
SCO3913	-2.86	0.01	hypothetical protein
SCO3964	-1.72	0.11	possible integral membrane protein
SCO4253	-4.31	0.00	conserved hypothetical protein
SCO4541	-1.95	0.06	possible membrane protein
SCO4984	-1.90	0.06	probable aminotransferase
SCO5039	-2.50	0.02	possible penicillin-binding protein
SCO5247	-2.09	0.05	possible deaminase
SCO5285	-2.47	0.02	lon, ATP-dependent protease
SCO5354	-1.63	0.13	thrA, homoserine dehydrogenase
SCO5457	-2.25	0.03	possible lipoprotein
SCO5540	-1.91	0.07	cvnA2, unknown
SCO5553	-1.80	0.10	leuC, 3-isopropylmalate dehydratase large subunit
SCO5617	-2.66	0.02	unknown prophage gene
SCO6142	-1.80	0.10	Unknown
SCO6462	-1.63	0.13	ogt2, methylated-DNA-protein-cysteine methyltransferase
SCO6587	-2.07	0.04	probable dehydrogenase
SCO6773	-1.54	0.13	possible secreted peptidase
SCO6847	-2.76	0.01	Unknown

SCO6856	-3.03	0.01	Unknown
SCO7063	-1.70	0.11	conserved hypothetical protein
SCO7116	-1.63	0.13	conserved hypothetical protein
SCO7252	-1.81	0.07	possible regulatory protein
SCO7304	-2.25	0.03	Unknown
SCO7354	-3.57	0.00	Unknown
SCO7361	-3.31	0.00	possible DNA-binding protein
SCO7458	-1.50	0.13	possible membrane protein
SCO7772	-1.92	0.07	unknown, length 194 aa
SCO7844	-1.74	0.11	possible DNA-binding protein
