# Supplementary material for: Development and application of versatile high density microarrays for genome-wide analysis of Streptomyces coelicolor: characterization of the HspR regulon
Source: Genome Biol. 2009 Jan 16;10(1):R5. doi: 10.1186/gb-2009-10-1-r5 (PMC2687793; doi:10.1186/gb-2009-10-1-r5)
Supplement: Additional data file 7 — Motif identified from MEME analysis of the HspR targets identified with the Sco-Chip2-v2 array. [file gb-2009-10-1-r5-S7.pdf]

Additional File 7

MEME alignment of upstream regions of putative targets of HspR as identified by Sco-Chip^2-v2

NAME	STRAND OF MOTIF DETECTION	START POSITION IN GENOME OF SITE DETECTED	P-VALUE	SITES		
tRNA_GLU	+	6049648	2.53e-15	CTCAAGGCGG	**TAGCGCCGGTTCGAATCCGGTCGGGGGTAC**	TCGAAGTCCA
tRNA_GLU	+	6049438	2.53e-15	CTCAAGGCGG	**TAGCGCCGGTTCGAATCCGGTCGGGGGTAC**	GAACTGGTCT
tRNA_GLU	+	6049303	2.53e-15	CTCAAGGCGG	**TAGCGCCGGTTCGAATCCGGTCGGGGGTAC**	AGATCCTTCC
SCO3671	-	4050797	2.61e-11	TCATGCACAC	**TTGAGCCTGTTCCACTCAAGTCAGCTGGAG**	GTTATTCACC
tRNA_GLN	+	6049192	2.65e-10	CTGGTTCAGT	**TAGTCTAGGTTCGAGTCCTGGTAGGCCAGC**	TCGCAGAGCT
tRNA_GLU	+	6049530	7.19e-10	CTGGTTCAGT	**TAGTCTAGGTTCGAGTCCTGGTAGACCAGC**	TCGGATCTGC
SCO5285	-	5756784	9.53e-10	GGCAGTCAAG	**TTGAGTTACATCGACTCAATGAAAGGGAGC**	CCGGGATTGT
SCO3671	-	4050745	7.44e-09	CGGATAAGAG	**TTGAGTCCGCTCGACTCACCTCTGTTGACC**	CATCGCCGGG
SCO3661	+	4040399	2.21e-08	GTACACAAAG	**TTGAGTCAGGGCCACTCAAGGAGTCGGGCG**	CACTTCTCCG
rRNA_100034	+	3300027	2.21e-08	TTCATCGGGA	**GAGTGCTTCGTCGAATTCGTTGATTCGGAG**	AAGCTGGTAG

For the target regions identified by Sco-Chip^2-v2 there were clear peaks of enrichment that indicated binding of HspR to the following targets: *SCO5285* (*lon*) (highest peak in Figure 3C), *SCO3671* (*dnaK*) (highest peak in Figure 3A), *SCO3661* (highest peak in *clpB*) (Figure 3B), rRNA_100034 (Figure 3D), tRNA_GLN (highest peak in Figure 3E) and tRNA_GLU (second highest peak in Figure 3F). Subsequently, the upstream regions (300 nt upstream of annotated start (of CDS or of mature stable RNA) and 200 nt downstream of the same annotated start in correct transcriptional orientation (as annotated)) for these target genes were taken and submitted to the MEME server [68, 69] for alignment. Default settings were used except for: minimum width of the motif to search for was set to 5 nt; maximum width to 30 nt; maximum number of sites to find was set to 10 and any number of repetitions were allowed.
